# Benchmarking inverse folding models for antibody CDR sequence design

**DOI:** 10.1371/journal.pone.0324566

**Published:** 2025-06-04

**Authors:** Yifan Li, Yuxiang Lang, Chenrui Xu, Yi Zhou, Ziwei Pang, Per Jr. Greisen

**Affiliations:** 1 BioMap Research, Haidian District, Beijing, China; 2 BioMap Research, Palo Alto, California, United States of America; National University of Singapore, SINGAPORE

## Abstract

Antibody-based therapies are at the forefront of modern medicine, addressing diverse challenges across oncology, autoimmune diseases, infectious diseases, and beyond. The ability to design antibodies with enhanced functionality and specificity is critical for advancing next-generation therapeutics. Recent advances in artificial intelligence (AI) have propelled the field of antibody engineering, particularly through inverse folding models for Complementarity-Determining Region (CDR) sequence design. These models aim to generate novel antibody sequences that fold into desired structures with high antigen-binding affinity. However, current evaluation metrics, such as amino acid recovery rates, are limited in their ability to assess the structural and functional accuracy of designed sequences. This study benchmarks state-of-the-art inverse folding models—ProteinMPNN, ESM-IF, LM-Design, and AntiFold—using comprehensive datasets and alternative evaluation metrics like sequence similarity. By systematically analyzing recovery rates, mutation prediction capabilities, and amino acid composition biases, we identify strengths and limitations across models. AntiFold exhibits superior performance in Fab antibody design, whereas LM-Design demonstrates adaptability across diverse antibody types, including VHH antibodies. In contrast, models trained on general protein datasets (e.g., ProteinMPNN and ESM-IF) struggle with antibody-specific nuances. Key insights include the models’ varying reliance on antigen structure and their distinct capabilities in capturing critical residues for antigen binding. Our findings highlight the need for enhanced training datasets, integration of functional data, and refined evaluation metrics to advance antibody design tools. By addressing these challenges, future models can unlock the full potential of AI-driven antibody engineering, paving the way for innovative therapeutic applications.

## Introduction

Antibody-based therapies have revolutionized modern medicine, offering effective treatments for diseases ranging from cancer (e.g., pembrolizumab [1]), autoimmune disorders (e.g., infliximab [2]), hemophilia A (e.g., emicizumab [3] or mim8 [4]), and infectious diseases (e.g., palivizumab [5]). As demand grows for antibodies with enhanced functionality and tailored specificity, the ability to design novel antibodies rapidly and possess the desired properties is increasingly critical for advancing next-generation biologics. Single-domain antibodies, such as VHHs, offer unique advantages due to their small size, stability, and ability to access previously undruggable targets [6]. Their therapeutic potential is evident in the approval of drugs like caplacizumab (Cablivi®) for aTTP [7], ciltacabtagene autoleucel (Carvykti™) for multiple myeloma [8], ozoralizumab for rheumatoid arthritis [9], and envafolimab for various cancers and hepatitis B [10].

Artificial intelligence (AI) holds tremendous potential to accelerate this process, providing innovative tools to tackle the challenges of antibody engineering. Among these tools, antibody sequence design models, also known as “inverse folding” methods, have demonstrated significant progress in generating novel sequences that fold into desired structures with high antigen-binding affinity [11–14].However, the rapid development of these methods necessitates careful and unbiased benchmarking to understand their respective strengths and limitations. A comprehensive evaluation is essential to guide researchers in selecting the most appropriate tool for a given design task and to identify areas where further development is needed. Direct comparison of these methods can be challenging due to variations in training data, architecture, and evaluation metrics used in their original publications. Therefore, a standardized and rigorous benchmarking approach is crucial for objective assessment.

**Fig 1:**
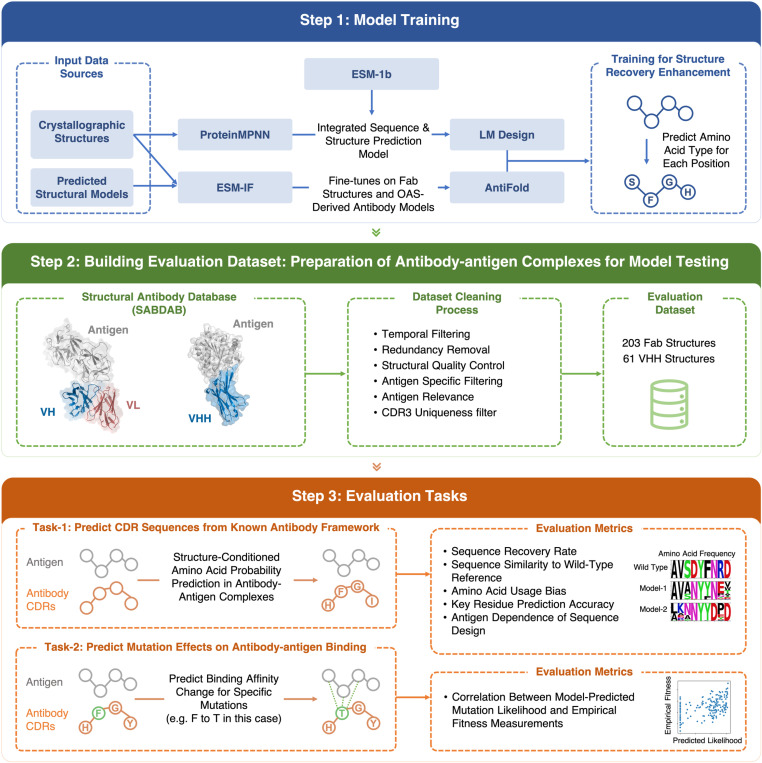
Overview of antibody inverse folding model benchmarking and evaluation framework. This schematic delineates our systematic framework for evaluating and benchmarking antibody inverse folding models for complementarity-determining region (CDR) sequence design. **Model Training (Blue Panel).** We evaluate four distinct inverse folding architectures with heterogeneous training paradigms. ProteinMPNN was trained exclusively on high-resolution experimentally determined crystallographic protein structures (<3.5Å). ESM-IF incorporates both crystallographic structures and AlphaFold2-predicted structural ensembles in its training corpus. LM Design integrates ProteinMPNN’s structural modeling capabilities with the ESM-1b protein language model, which was pre-trained on 250 million protein sequences, for sequence optimization. AntiFold was fine-tuned from ESM-IF using experimentally solved Fab structures from SAbDab (n = 2,074) and computationally predicted Fab structures from OAS (n = 147,458). All models were optimized to maximize native sequence recovery accuracy for CDR prediction, quantified via perplexity and amino acid recovery metrics. **Benchmark Dataset (Green Panel).** Our evaluation dataset was curated from the Structural Antibody Database (SAbDab) through stringent filtration criteria including resolution thresholds (<3.5Å), conformational quality assessment, redundancy elimination (95% sequence identity cutoff) (see Methods for comprehensive protocol). The resultant dataset comprises 203 Fab structures and 61 VHH (single-domain antibody) structures, providing statistically robust representation for model evaluation. **Evaluation Metrics (Orange Panel).** We quantitatively assess model performance in two biophysically relevant domains: 1. CDR Sequence Prediction: Evaluates design accuracy via sequence recovery, perplexity, and biochemical property conservation when predicting CDR sequences conditioned on antibody framework structures with explicit consideration of antigen-binding interface constraints. 2. Mutation Effect Prediction: Quantifies correlation (Spearman’s ρ) between model-predicted substitution probabilities and experimentally determined ΔΔG values or deep mutational scanning data to assess the models’ capacity to identify functionally critical residues.

In this study, we address this critical need by systematically benchmarking several widely used inverse folding models for antibody design: ProteinMPNN [11], ESM Inverse Folding (ESM-IF) [12], LM-Design [13], and AntiFold [14]. Some important features of these models are summarized in Table 1 and Fig 1. We focus on these specific models due to their public availability, widespread adoption within the research community, and diverse underlying architectures, allowing for a broad exploration of the current landscape of inverse folding methodologies. By highlighting the strengths and weaknesses of these algorithms across a range of relevant metrics, our study provides valuable insights into their robustness and offers a foundation for future improvements in antibody engineering tools. We initially evaluated AbMPNN [15], a model that shares similarities with AntiFold in terms of training data and strategy. However, we focused our benchmarking efforts on AntiFold, as its authors reported superior performance compared to AbMPNN, a finding that is consistent with our own results (S4 and S5 Tables in S1 File).

**Table 1:** Important training and inference features of evaluated models.

	ProteinMPNN	ESM-IF	LM Design	AntiFold
Training and fine-tuning method	Trained with a message-passing neural network	Trained with a transformer architecture with Geometric Vector Perceptron (GVP) layers	Combined protein language models with a structure encoder	Fine-tuned from ESM-IF1
Training data	High-resolution protein structures from the Protein Data Bank	Experimentally solved and predicted structures	High-resolution protein structures from the Protein Data Bank	Experimentally solved Fab structures from SAbDab and predicted Fab structures from OAS. VHH was not included in the training data.
Inference method/speed	Autoregressive/Low	Autoregressive/Low	Non-autoregressive/High	Non-autoregressive/High

Historically, inverse folding models have been evaluated using amino acid recovery rates, which measure how accurately a model reproduces the exact native sequences of the designed regions [11,13–16]. State-of-the-art (SOTA) models have demonstrated remarkable progress, achieving amino acid recovery rates exceeding 50% for the Complementarity-Determining Regions (CDRs)—key regions for antigen recognition and binding [13–15].

However, amino acid recovery rates alone provide an incomplete assessment of model performance. First, recovery metrics penalize predictions that closely resemble native residues but differ by minor substitutions, such as lysine (K) to arginine (R), even though such substitutions often preserve structural and functional properties. Secondly, recovery metrics fail to account for the strong amino acid composition biases in CDR regions, such as the overrepresentation of glycine (G), serine (S), and tyrosine (Y). Models may exploit these biases to achieve high recovery rates without capturing the structural or functional constraints critical to antigen recognition. Finally, high-affinity binding often relies on a subset of critical residues within the CDRs, making it more relevant to evaluate a model’s ability to recover these key residues rather than all residues indiscriminately.

To overcome these limitations, we adopt sequence similarity as an alternative evaluation metric (Fig 1). Sequence similarity accounts for physicochemical properties, such as charge and hydrophobicity, allowing a more nuanced assessment of how well a designed sequence preserves native-like functionality. For instance, substitutions like lysine (K) to arginine (R) maintain positive charge and binding potential, making sequence similarity more reflective of practical design requirements. Furthermore, this metric prioritizes the accurate recovery of residues critical for antigen-antibody interactions, which are pivotal for therapeutic applications.

Some studies have also evaluated inverse folding models by assessing their ability to predict changes in affinity resulting from mutations [17]. However, these evaluations often involve limited datasets and methods. In this study, we address this limitation by systematically benchmarking the models using a comprehensive collection of deep mutational scanning datasets, but also to address the robustness of the different algorithms for antibody design.

## Methods

### Evaluation tasks for antibody inverse folding models

To assess the performance of inverse folding models, we employed two key benchmarking tasks (Fig 1):

#### 1. Antibody sequence design.

Each model was tasked with designing CDR sequences for given antibody-antigen complex structures. We use Fab to refer to two chain antibody binding fragments across the whole manuscript. For Fab antibodies, all six CDRs, three from the heavy chain (CDRH1(H1), CDRH2(H2), CDRH3(H3)) and three from the light chain (CDRL1(L1), CDRL2(L2), CDRL3(L3)) were designed. For VHH antibodies, only the three heavy chain CDRs (H1-H3) were included.

#### 2. Mutation effects on antibody affinity: Predictions and correlations.

Single-site mutagenesis was performed for all CDR residues, and models were used to calculate log-likelihood scores for each mutated sequence. These scores were then correlated with experimentally measured changes in binding free energy (ΔΔG) to evaluate the models’ ability to predict mutational effects.

### Sequence design evaluation dataset

We employed the Structural Antibody Database (SAbDab) [18,19] as the foundation for our evaluation dataset and applied rigorous filtering criteria to ensure data quality and relevance (Fig 1). These criteria included recency, sequence redundancy, structural quality, antigen relevance, and CDR3 uniqueness (summarized in Table 2). By applying these steps, we curated a high-quality dataset comprising diverse and recent antibody-antigen complexes suitable for benchmarking sequence design models. This filtering yielded 203 Fab and 61 VHH antibody structures (see S2 and S3 Tables in S1 File for PDB IDs and chains).

**Table 2:** Filtering criteria applied to the SAbDab dataset for sequence design evaluation.

Filtering criteria	Description	Rationale
Timeline Filtering	Included entries released after January 1, 2023	Aligns with training data of the latest inverse folding models
Sequence Redundancy	Excluded sequences with >95% identity to entries before January 1, 2023	Reduces data leakage and ensures diversity.
Structural Quality	Removed structures with resolutions >3.5 Å or missing residues (e.g., those that cause broken chains in the crystal structure)	Ensures high-quality templates for modeling and prevents errors in sequence design models that rely on complete structures.
Antigen Filtering	Excluded entries lacking an antigen or with multiple interacting chains	Focuses on antibodies with well-defined antigen interactions.
Antigen Relevance	Excluded entries with coronavirus antigens	Mitigates overrepresentation of coronavirus related entries (~30% of the population);
CDR3 Uniqueness	Retained unique CDR3 sequences; removed antibodies with identical CDR3 sequences.	CDR3 is highly diverse and critical for antigen binding; redundant sequences reduce dataset value

### Sequence design protocol

For sequence design, PDB files were preprocessed to retain only the Fv region (for Fab) or the VHH chain, along with the antigen chain. Complexes with multiple antigen chains were excluded. CDR regions were defined using the Chothia numbering scheme [20] with the AbNumber tool [21]. Unless otherwise noted, we generated 100 designed sequences for all CDR regions per sample, using a sampling temperature of 0.2. The design process was conditioned on the complex structure, incorporating both antibody and antigen coordinates as input. Antigen sequences and antibody framework sequences were not included in the input due to limitations in ESM-IF and AntiFold and also because preliminary experiments demonstrated that conditioning on antigen sequences had minimal effect on sequence recovery (see S12 Fig in S1 File). For antigen-free design, the antigen chain was removed from the input PDB file. For relaxed structure design, PDBs underwent three rounds of Rosetta FastRelax with coordinate constraints (weights = 1.0) [22–24] (see Supplementary Method for detailed relaxation *xml* file). Sequence recovery and similarity were calculated for each CDR region and PDB file. The running environment for all models was as follows: 0.5 NVIDIA A100 GPU, 5 CPU cores, 50 GB RAM.

### Sequence design evaluation metrics

To quantify the quality of the sequence designs, we analyzed design identity, design similarity, amino acid bias, and design refolding consistency for the evaluated models. Design identity is calculated as the sum of all perfectly recovered residues divided by the total number of designed residues. Design similarity accounts for the physicochemical similarities between amino acids. We used the BLOSUM62 [25] substitution matrix to quantify the similarity between designed and wild-type (WT) residues. Design similarity is calculated as the sum of BLOSUM62 scores for all designed residues, normalized by the sum of BLOSUM62 scores for the WT residues.

The equation for design similarity is calculated as Equation (1):


$$DS=\frac{\sum iBLOSUM62\left({designAA}_{i},{wtAA}_{i}\right)}{\sum iBLOSUM62\left({wtAA}_{i},{wtAA}_{i}\right)}$$ (1)

*DS* represents the design similarity score. *BLOSUM62(residueA,residueB)* denotes the BLOSUM62 substitution score between residue A and residue B; *designAA*_*i*_ is the designed amino acid at position i; *wtAA*_*i*_ is the wild-type amino acid at position i.

To analyze the consistency with the protein folding model, we utilized the designed antibody sequences to perform protein refolding and calculated the root mean square deviation (RMSD) between the refolded structures and the template. Protein refolding was carried out using the Boltz-1 [26], with only the designed antibody sequences included. The antigen was not included in the antibody-antigen complex prediction due to the low success rate observed even with state-of-the-art complex prediction models. To mitigate the computational expense of multiple sequence alignment (MSA) searches and reduce the influence of MSA on the prediction, we employed a consistent MSA across all designed sequences for a given PDB template. Specifically, we constructed the MSA by searching for homologs using the wild-type sequence from the PDB. During refolding of designed sequences, we replaced the first (query) sequence in the MSA with the designed sequence, while keeping the rest of the alignment unchanged. For each PDB template, we sampled five sequences for folding. Two folding structures were generated for each sequence, and the one with the highest confidence score was selected for RMSD calculation. For each structure, we performed structural alignment based on the entire antibody region, that is, the heavy chain for VHH antibodies and the combined heavy and light chains for Fab antibodies. After alignment, we calculated RMSD values exclusively for the designed CDR regions, excluding the framework residues from the analysis.

To analyze the prediction accuracy for each residue, we generated logo plots according to a method depicting both residue frequency and conservativeness [27]. The height of each residue is made proportional to its frequency in the design, and the letters are sorted so the most common one is on top. The height of the entire stack is then adjusted to signify the information content of the sequences at that position, which is represented by the decrease in uncertainty as the binding site is located. The size of each residue printed in a logo is determined by multiplying the frequency of that base by the total information at that position. The calculation can be found in the work [27].

It is worth noting that some studies have utilized Rosetta energy scores (e.g., total_energy, dG_separated) to evaluate the quality of designs. However, we did not include this metric in our evaluation, as our preliminary analysis indicated only minor differences in energy scores between models. Additionally, the interactions present in the crystal structures themselves and the relaxation protocol appeared to have a greater influence on the energy terms.

### Categorization of CDR residues by location and functional role

To investigate the relationship between residue location, function, and design accuracy, we categorized CDR residues based on their relative solvent accessible surface area (SASA) and predicted contribution to antigen binding.

#### SASA calculation and buried residues.

SASA was calculated for each residue in the unbound antibody (apo) format using the Shrake-Rupley algorithm [28,29]. Residues with a relative SASA below 20% were classified as “buried”, indicating limited solvent exposure.

#### Predicting binding contributions and key interactions.

We employed a Rotamer Density Estimator (RDE) based mutation effect prediction model to predict the contribution of each residue to the binding [30]. This model was trained to predict the probability density of protein side-chains and to estimate the mutational ∆∆G (the change in binding free energy with a specific mutation in the binding region) with the representations learned from the pre-trained model. To estimate the contribution of each residue for antigen binding, we performed an *in silico* alanine scanning to all residues in the CDR regions. More specifically, we mutate the native residue to alanine for all CDR residues and calculate the ΔΔG for the mutation. A higher ΔΔG indicated a larger drop of binding affinity with the mutation. We define residues with a ΔΔG greater than 1.5 kcal/mol upon alanine substitution as “key interaction” residues, representing around 5% of all the residues in the CDR regions.

#### Surface contact residues.

Residues located within 4 Å of any antigen atom but not classified as key interaction residues were categorized as “surface contact” residues. These residues are solvent-exposed and in contact with the antigen but are not predicted to make major energetic contributions to binding.

This categorization scheme allowed us to assess the models’ performance in designing sequences for residues with distinct structural and functional roles.

### Mutation scoring dataset

To benchmark the prediction of mutational effects, we curated datasets based on three criteria. First, the datasets must measure the impact of mutations on antibody-antigen binding, excluding any data related to antigen mutational scanning or mutations not associated with antibody binding complex. Second, each dataset must contain at least 20 mutation data points in the heavy chain to ensure that the calculated correlations have sufficient statistical power. Third, the dataset focuses only on heavy chain mutations with a fixed light chain, since a combination of heavy and light mutations will complicate the task. The datasets we collected include:

1. SKEMPI2 dataset [31]: contains mutations and relevant ddG. We could find two complex structures that have more than 20 mutation data points and calculate per-structure correlation of ddG and log likelihood for these structures. One of the structures (PDB ID: 1VFB) involves the anti-hen egg white lysozyme (HEL) antibody D1.3 bound to HEL. The data were generated by two independent studies conducted in the same laboratory, performing alanine scanning mutagenesis on the antibody and measuring the affinities [32,33]. The other complex (PDB ID: 1MHP) involves an antibody fragment bound to the I-domain of the integrin VLA1 [34].
2. Affinity-matured influenza broadly neutralizing antibodies (bnAbs) CR9114 and CR6261 [35]: The profiled mutational landscape of CR9114 includes all possible combinations of 16 substitutions, whereas that of CR6261 includes all possible combinations of 11 substitutions, totaling 65,536 and 2048 variant antibody sequences, respectively.
3. Shira Warszawski performed saturation mutagenesis for all CDR residues for an anti-lysozyme antibody (PDB ID: 1MLC), and used yeast display and deep sequencing to estimate the affinity of each mutation [36].

### Data and code availability

All data and code utilized in this study, including scripts for executing inverse folding models, PDB files from the evaluation dataset, raw designed sequences, scripts for data processing and analysis, and Rosetta XML files for structure relaxation, are available at: https://github.com/biomap-research/InverseFoldingEvaluation

## Results

### Benchmarking antibody design models: recovery rates and similarity across CDRs

#### Performance across fab antibodies.

**Fig 2:**
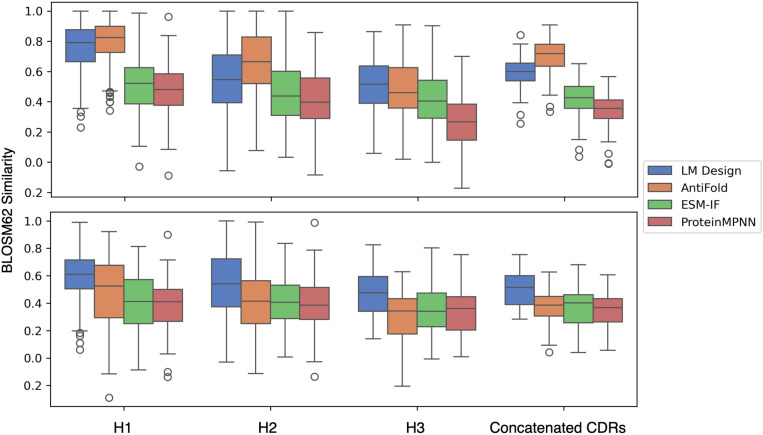
Design methods compared for Fab (upper panel) and VHH (lower panel) using BLOSUM62 similarity scores. Models were evaluated on 203 Fab crystal structures and 61 VHH crystal structures from SAbDab. Analyses were restricted to the H1, H2, and H3 regions, as defined by the Chothia numbering scheme.

**Fig 3:**
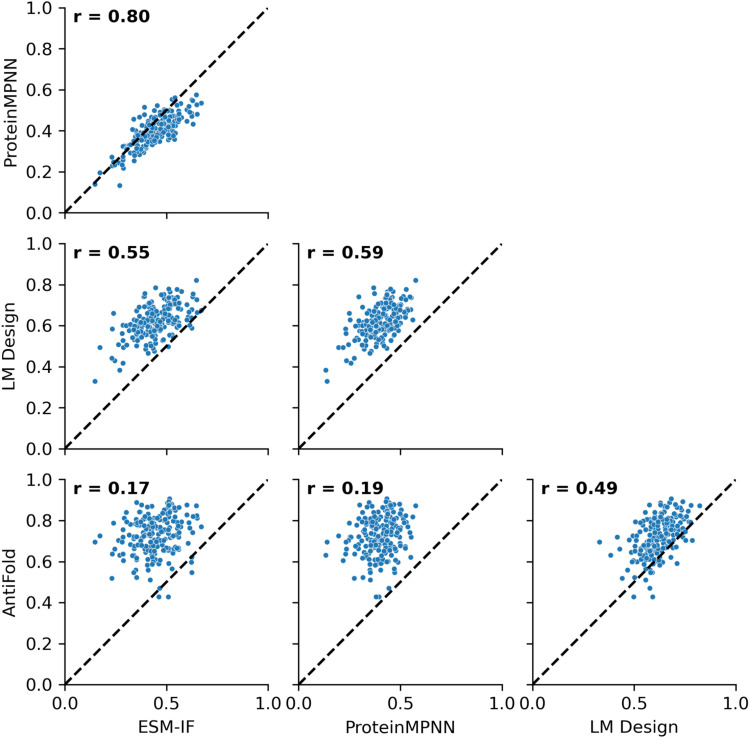
Pairwise correlation of recovery rates for concatenated CDR sequences across fab PDB structures using various models. Scatter plots showing the pairwise correlation of recovery scores for concatenated CDR sequences across Fab structures in the benchmarking dataset. Each dot represents the recovery score for an individual PDB structure. Recovery or similarity scores were computed for all concatenated CDR regions. Pearson correlation coefficients (r values) are indicated in each plot, quantifying the strength of the relationship between models.

We evaluated the sequence recovery and similarity of CDRs designed by each model for Fab antibodies. The models ranked in performance as follows: AntiFold > LM-Design > ESM-IF> ProteinMPNN (Figs 2 and 3, and see S6 Table in S1 File for statistical significance). Notably, AntiFold consistently outperformed the other models, achieving higher recovery rates and sequence similarity across all CDR regions except for H3 (see S1 Fig and S4 Table in S1 File for detailed data). LM-Design demonstrated robust performance, particularly in recovering residues within canonical CDR regions, where it surpassed ESM-IF and ProteinMPNN. However, its recovery rates for concatenated CDR regions were slightly lower than AntiFold, which excelled in capturing the structural diversity of these regions. These findings underscore AntiFold’s ability to design sequences that align closely with native structures.

#### Performance across VHHs.

For VHH antibodies, the performance ranking shifted: LM-Design > AntiFold ≈ ESM-IF ≈ ProteinMPNN (Fig 2 and see S7 Table in S1 File for statistical details). LM-Design achieved the highest recovery and similarity scores across all CDR regions, highlighting its adaptability to the compact and simplified architecture of VHH antibodies. In contrast, AntiFold, which performed exceptionally well for Fab antibodies, showed reduced efficacy for VHH sequences. This discrepancy likely stems from its training data, which lacked substantial representation of VHH antibodies. Despite this limitation, AntiFold maintained competitive performance in H1 and H2, demonstrating some generalizability across antibody types (S2 Fig and S5 Table in S1 File).

#### CDR-specific trends.

The recovery rates and similarity scores varied significantly across the six CDR regions in Fab antibodies and three in VHH antibodies. AntiFold showed remarkable performance in H1 and H2 for both Fab and VHH, reflecting its strength in designing residues within relatively conserved regions. However, its performance for H3 was mixed, excelling in Fab antibodies but lagging in VHH sequences.

LM-Design’s performance was more uniform across all CDRs, particularly excelling in the highly variable H3 of VHH antibodies. ESM-IF and ProteinMPNN showed comparable recovery rates. These trends are summarized in Figs 2, S1, S2 and S4 and S5 Tables in S1 File.

#### Refolding of designed sequences.

To assess the consistency of the designed sequences with structure prediction models, we performed sequence sampling and refolding using Boltz-1, followed by alignment of the refolded structures with the design template. The root mean square deviation (RMSD) was then calculated for the complementarity-determining regions (CDRs). No significant differences were observed between the various models (S13 Fig in S1 File). It is important to note that even state-of-the-art folding algorithms face challenges in accurately predicting the complex formed by an antibody and its antigen, or even the isolated CDR conformation. As such, the refolding model itself may introduce considerable noise, limiting its ability to reliably assess folding accuracy.

### Analysis of amino acid prediction accuracy

**Fig 4:**
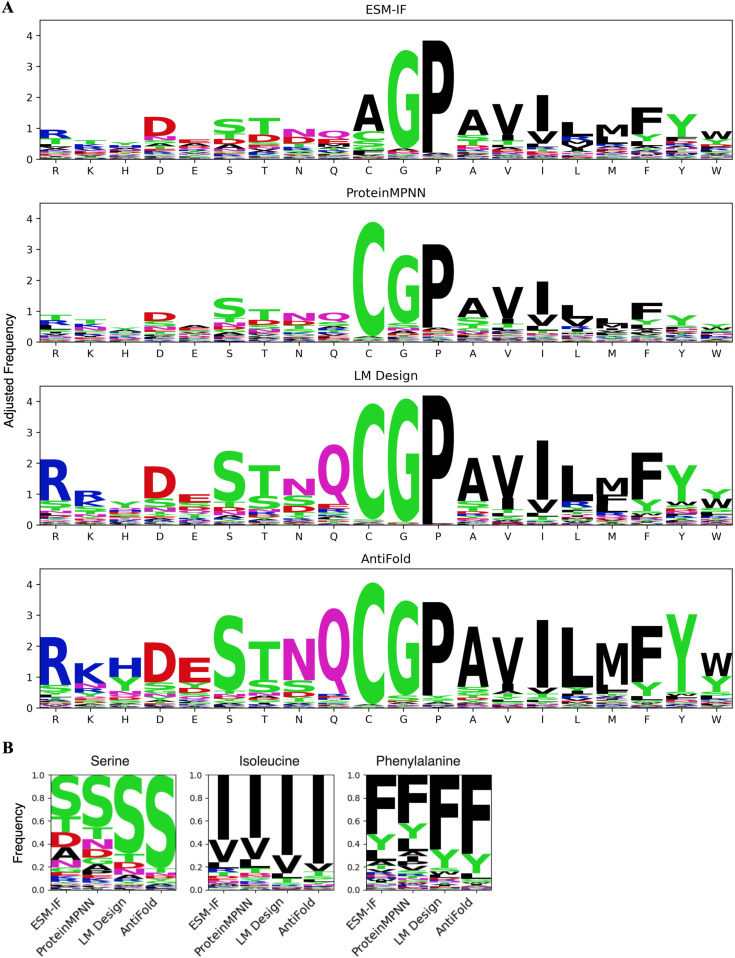
Amino acid prediction accuracy for fab design. (A) Logo plots showing the predicted amino acid frequencies for each wild-type (WT) residue in the CDRs of Fab structures. WT residues are ordered by descending frequency. The height of each letter indicates the relative frequency of predicted amino acids, highlighting each model’s substitution tendencies. The overall height of each column indicates the conservativeness of the design for each type of residue. The size of each residue printed in a logo is determined by multiplying the frequency of that base by the total information at that position (See S11 Fig in S1 File for a corresponding analysis of VHH CDR designs). (B) Residue-specific prediction analysis for three representative amino acid types (Serine (S), Isoleucine (I), and Phenylalanine (F)) across different models. Amino acid predictions are ranked by frequency, highlighting model preferences for substitutions at specific positions.

To assess the models’ ability to recover specific amino acid types within the CDRs, we analyzed the prediction accuracy for each residue type and plotted the amino acid frequencies and conservativeness in Fig 4 [27]. Overall, AntiFold demonstrated the highest recovery rates, followed by LM Design, and then ESM-IF and ProteinMPNN, which showed comparable performance (Figs 4A, S10 and S11 in S1 File). Notably, AntiFold outperformed LM Design in recovering almost every amino acid type, except proline and glycine.

Further examination revealed an interesting trend across all models. Even when incorrect predictions occurred, the substituted residues often exhibited similar physicochemical properties to the WT residues. Common substitutions included those within the following groups: lysine (K)/ arginine (R), threonine (T)/ serine (S), and tryptophan (W)/ phenylalanine (F)/ tyrosine (Y). We selected three representative amino acid types—S, isoleucine(I), and F — and calculated the frequency of predicted types (Figs 4B and S6 in S1 File). It is evident that for all models, the most frequent predictions correspond to the native residues, while the second most frequent predictions exhibit chemical properties closely resembling those of the native residue (e.g., Y for F, valine(V) for I, and T for S). This observation suggests that the models have learned to recognize and preserve key amino acid properties during sequence design, even if they do not always predict the exact native residue.

### Analysis of amino acid frequency bias in designed sequences

**Fig 5:**
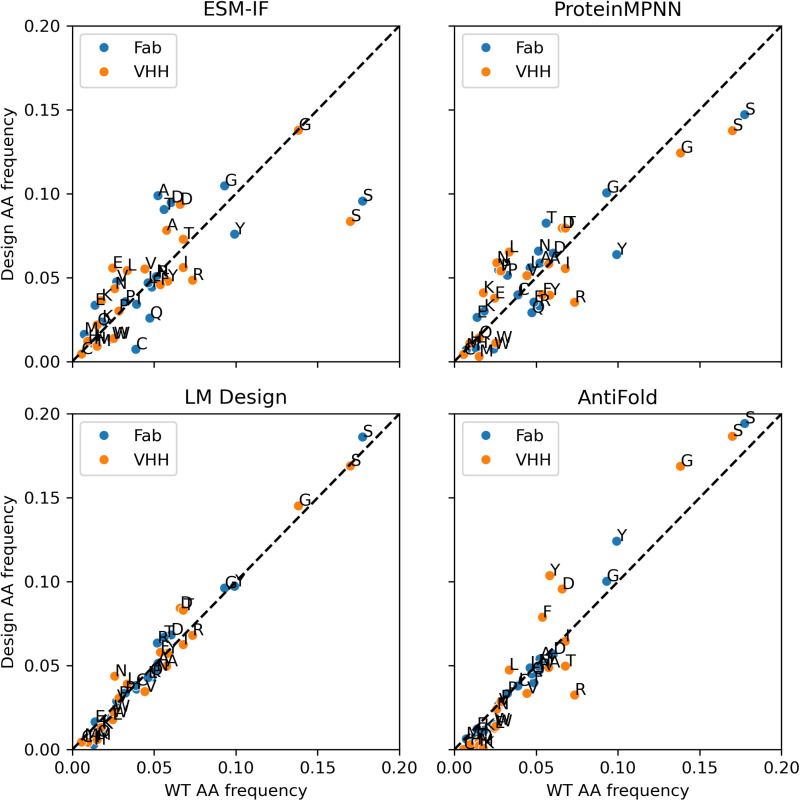
Amino acid frequency comparison between designed and wild-type sequences. Scatter plots comparing the amino acid frequencies of designed sequences (y axis) to their wild-type (WT) counterparts (x axis) for Fab (blue circles) and VHH (orange circles). The frequencies were calculated by counting all WT and designed residue types across the benchmarking dataset. The diagonal dashed line indicates a perfect match between designed and WT frequencies.

To assess whether the models accurately capture the natural amino acid distribution within CDRs, we analyzed the frequency of each residue type in the designed sequences (Fig 5). For Fab antibody design, LM Design and AntiFold exhibited a stronger correlation with natural amino acid frequencies compared to ProteinMPNN and ESM-IF. This suggests that LM Design and AntiFold more effectively mirror the observed biases in amino acid usage. For example, both models accurately captured the overrepresentation of phenylalanine and serine in CDR regions, which was less pronounced in the sequences generated by ProteinMPNN and ESM-IF.

In contrast, for VHH antibody design, LM Design aligned closely with natural amino acid frequencies, whereas AntiFold deviated significantly. AntiFold exhibited a tendency to overrepresent high-frequency residue types in its designs (Figs 5 and S10 in S1 File). This discrepancy suggests potential differences in how AntiFold models Fab and VHH sequences. We hypothesize that AntiFold, trained predominantly on a Fab dataset, may attempt to approximate human IGHV3 sequences when designing VHH formats, as IGHV3 is the closest match in its training data. Supporting this hypothesis, AntiFold-designed VHH sequences exhibited amino acid biases similar to IGHV3 sequences, including a higher frequency of Y, D, F, G, and S, and a lower frequency of R and T. For details, refer to S10 Fig in S1 File and the findings presented in Gordon et al. [37].

### Sequence design performance by residue location and role

Recognizing that amino acid residues in different regions of an antibody play distinct functional roles, we evaluated the models’ ability to design sequences based on residue location and role in antigen binding. We categorized CDR residues into three groups:

- Buried: Residues with minimal solvent exposure in the unbound antibody (apo) format (relative SASA < 20%).
- Key Interaction: Residues identified as critical for antigen binding based on their RDE score, a measure of the impact of alanine mutations on binding affinity (predicted ΔΔG greater than 1.5 kcal/mol upon alanine substitution).
- Surface Contact: Residues located on the antibody surface that contact the antigen (within 4 Å of any antigen atom) but are not classified as key interaction residues.

Detailed criteria for these classifications are provided in the Methods section.

**Fig 6:**
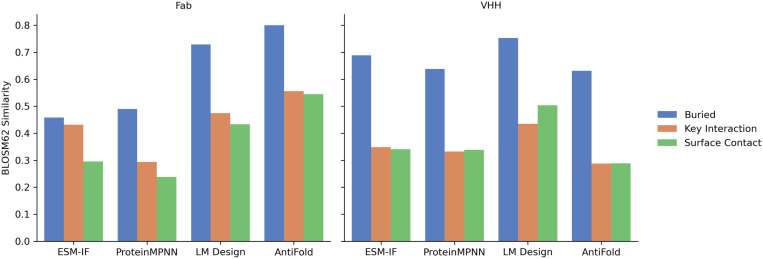
Sequence design similarity for residues in different functional regions of VHH (left) and Fab (right). BLOSUM62 similarity scores (y-axis) were calculated for different sequence design models (x-axis). Residues were categorized into three functional groups—buried, key interaction, and surface contact—based on their structural location and functional role within the complex. Sequence similarity was assessed by comparing designed residues in each region to their corresponding wild-type residues. This analysis highlights model performance across distinct functional regions.

We then calculated the design similarity and recovery rates for each residue group (Fig 6). Across all models and antibody types, buried residues exhibited the highest design similarity, likely due to their greater conservation compared to surface residues. For Fab antibodies, AntiFold and LM Design showed significantly higher recovery rates for buried residues than ESM-IF and ProteinMPNN.

Interestingly, performance differences between models were less pronounced for key interaction residues. Notably, ESM-IF achieved similarity values for key interaction residues comparable to buried residues. Moreover, ESM-IF showed the largest difference in similarity between key interaction residues and surface contact residues. These findings suggest that ESM-IF may be particularly sensitive to the structural context of the antigen-binding interface.

### Impact of antigen structure on sequence design

**Fig 7:**
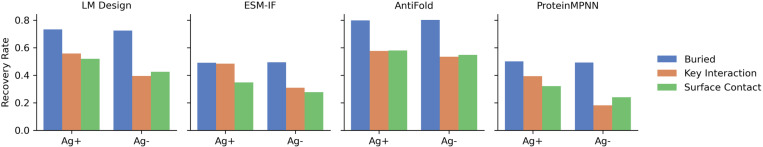
Sequence design recovery rates with antigen (Ag+) and without antigen (Ag-). Recovery rates (y-axis) for designed sequences were analyzed under two conditions (x-axis): with antigen present (Ag+) and without antigen (Ag-), where the antigen chain was removed from the PDB structure. Residues were classified into three functional categories—buried, key interaction, and surface contact—based on their structural role. Bars represent recovery rates for residues in these regions across different design models (LM Design, ESM-IF, and AntiFold), illustrating the impact of antigen presence on sequence design accuracy.

To investigate the influence of the antigen’s structural context on the models’ sequence design capabilities, we compared sequence recovery rates with and without the antigen chain present in the input PDB structure (Figs 7 and S7 in S1 File). This allowed us to assess the extent to which each model relies on the antigen’s presence for accurate sequence design.

We found that LM Design, ProteinMPNN, and ESM-IF produced slightly lower sequence design similarities when the antigen was excluded, indicating that these models utilize antigen information to some extent. In contrast, AntiFold showed nearly identical design similarities regardless of the antigen’s presence, suggesting that it primarily relies on the CDR loop structure for sequence design.

To further explore this observation, we analyzed the recovery rates for different groups of residues (buried, key interaction, and surface contact) with and without the antigen present (Fig 7). As expected, the recovery rates for buried residues remained consistent regardless of the antigen’s presence. However, for key interaction and surface contact residues, the models exhibited varying degrees of dependence on the antigen structure. ESM-IF and ProteinMPNN showed a significant drop in recovery rates for these residues when the antigen was absent, highlighting its reliance on antigen information for designing interface residues. Conversely, AntiFold’s performance remained consistent, reinforcing the observation that it primarily designs sequences based on the antibody’s structure rather than its interaction with the antigen.

### Illustrative design examples and model-specific performance

**Fig 8:**
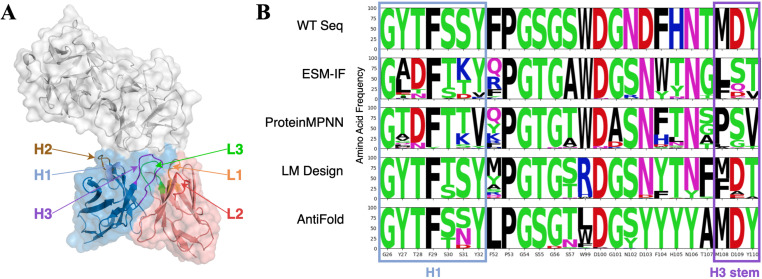
Analysis of CDR design and natural sequence distributions for Fab SylH3. (A) Structural representation of Ricin in complex with Fab SylH3 (PDB ID: 8TFL), highlighting the CDR regions indicated with different colors (H1, H2, H3, L1, L2, and L3). (B) Sequence logo plots of 100 heavy chain designs generated for PDB 8TFL using different models (ESM-IF, ProteinMPNN, LM Design, and AntiFold). Conserved regions (H1 region in blue box the H3 stem region in purple box) are highlighted, indicating sites of sequence constraint. Amino acid diversity and conservation patterns are illustrated for each model, showing deviations from wild-type sequences.

To gain deeper insights into the performance of different models, we analyzed the logo plots of several representative designs. PDB 8TFL, which depicts a neutralizing Fab fragment SylH3 bound to ricin toxin, serves as an illustrative example. We found that AntiFold and LM Design achieved nearly 90% recovery rates for H1, whereas ProteinMPNN and ESM-IF exhibited recovery rates of only around 40% (Fig 8 and S1 Table in S1 File). For H3 design, AntiFold and LM Design were more successful in accurately designing the stem portion compared to ProteinMPNN and ESM-IF. H1 and the stem of H3 are characterized by relatively conserved sequence patterns, and it is evident that AntiFold and LM Design have effectively learned these conserved patterns for antibody CDRs. For instance, in H1, the G-Y/F-T-F-S/T-S-Y sequence is one of the most frequent patterns [38], and both LM Design and AntiFold were able to replicate it.

Additionally, we observed that AntiFold achieved a much higher recovery rate for L2 compared to all other methods. For example, in the case of PDB 8TFL, AntiFold reached an 85% recovery rate, while the other methods hovered around 50% (S1 Table in S1 File). L2 has a limited number of canonical structures, and AntiFold has successfully learned the structure-sequence relationship for this region.

**Fig 9:**
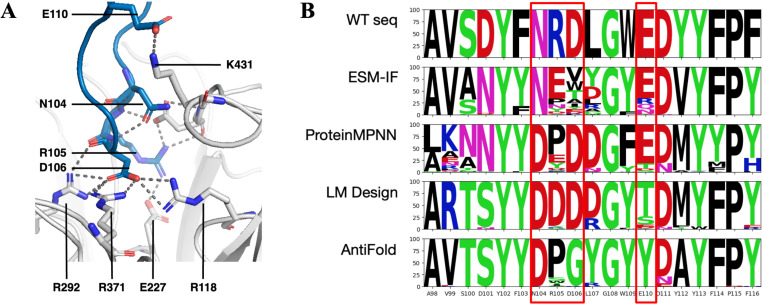
Analysis of antibody-neuraminidase interaction and heavy chain design. (A) Key interactions between the FNI9 antibody (shown in blue) and the neuraminidase catalytic site (shown in light gray). Highlighted residues represent critical binding interactions within the epitope emphasizing key interactions that contribute to antigen recognition and affinity. (B) Sequence logo plots showing the amino acid frequency distributions for 100 heavy chain designs of FNI9 in complex with N2 neuraminidase (PDB ID: 8G3V) generated using ESM-IF, ProteinMPNN, LM Design, and AntiFold. Key epitope-contacting residues, highlighted in red boxes, illustrate patterns of conservation and substitution across the designs, providing insights into sequence variability and structural constraints.

To further evaluate model performance, we investigated instances where models other than AntiFold excelled in H3 design (Fig 9). PDB 8G3V, featuring the FNI9 antibody complexed with neuraminidase, provides a representative example [39]. In this structure, H3 of FNI9 interacts with three highly conserved arginine residues within the neuraminidase catalytic site. Our analysis revealed that AntiFold did not effectively recapitulate these key binding interactions. Specifically, for the critical residues R105, D106, and E110, LM Design and ProteinMPNN successfully recovered D106, while ProteinMPNN and ESM-IF recovered E110. In contrast, AntiFold failed to generate sequences that maintained these interactions, indicating potential limitations in its ability to prioritize functionally critical residues within the H3 loop.

### Predicting the effects of mutations

Beyond sequence design, inverse folding models can also be employed to predict the effects of mutations on antibody binding affinity. By evaluating the log-likelihood of mutant sequences conditioned on the antibody-antigen complex structure, these models can provide insights into the functional consequences of amino acid substitutions.

To benchmark the models’ mutation prediction capabilities, we utilized two types of datasets:

1. SKEMPI2 Database: This database contains a curated set of experimentally determined changes in binding free energy (ΔΔG) for a variety of protein mutations. We chose two antibody-antigen complexes from this database with over 20 mutation data points each [32,34]. Refer to the method section for detailed description of the dataset identified.
2. Deep Mutational Scanning Data: These datasets provide comprehensive mutational landscapes for specific antibodies, typically generated through saturation mutagenesis or combinatorial mutations of CDR regions. The datasets include corresponding binding affinity measurements obtained via high-throughput screening methods. We incorporated several publicly available deep mutational scanning datasets for well-characterized antibodies [35,36].

**Fig 10:**
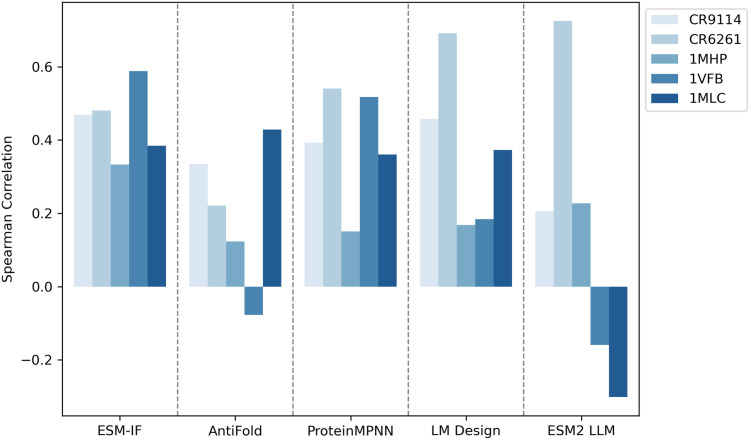
Spearman correlation between model predictions and experimental fitness scores. Spearman correlation coefficients were calculated between the log-likelihood scores predicted by different models and the experimentally measured fitness scores across five independent datasets (CR9114, CR6261, 1MHP, 1VFB, and 1MLC). In addition to ESM-IF, AntiFold, ProteinMPNN, and LM Design, a protein large language model ESM2 was included in the benchmark. The results highlight the variability in model performance across datasets and the impact of incorporating advanced language models into the evaluation.

We used the inverse folding models to compute the log-likelihood scores for each mutation in these datasets, conditioned on the corresponding template PDB structures. We then analyzed the Spearman correlation between the log-likelihood scores and the experimentally measured changes in binding free energy (ΔΔG) or affinity values. We also included additional language models ESM2 [40] in the benchmark (Fig 10).

Our analysis revealed that ESM-IF, ProteinMPNN, and LM Design consistently exhibited positive correlations between their log-likelihood scores and the experimentally measured mutation fitness. This suggests that these models can effectively capture the impact of mutations on antibody binding affinity. In contrast, AntiFold and other protein language models showed less consistent correlations, with greater fluctuations across datasets. Notably, ESM-IF demonstrated the highest average correlation across all datasets, indicating its superior performance in predicting the effects of mutations (Figs 10 and S9 in S1 File). This phenomenon likely stems from its design in the training and inference process. ESM-IF was trained exclusively on single chains, and for complex inference, it simply concatenates chains. As a result, ESM-IF may misinterpret complex interfaces as analogous to the cores of single-chain proteins during sequence design. This can cause the model to overemphasize mutations that disrupt interface interactions, treating them as though they were internal to a single chain rather than part of a multi-chain assembly. The ESM2 LLM exhibited a remarkable correlation with the CR6261 dataset, which features a diverse array of mutation combinations spanning both the CDR and framework regions. Notably, some of these combinations might negatively impact antibody stability, a factor the LLM seems adept at identifying and capturing [41]. However, it showed near-zero or even negative correlation for some dataset. ESM2 was trained on large-scale sequence databases and capture evolutionary signals across a broad range of proteins. While this approach is effective for many protein families, it may not be well-suited for ranking critical interactions for antibody binding.

## Discussion

### Common features and learned properties

Despite performance variations, our benchmark revealed common features among the evaluated inverse folding models. Notably, all models demonstrated an understanding of amino acid physicochemical properties, as evidenced by the confusion matrices. Amino acids with similar properties, such as the aromatic residues F/W/Y, the aliphatic residues I/V/L, and the negatively charged residues D/E, exhibited higher confusion rates within their respective groups. This indicates that the models have learned to associate these residues based on shared characteristics.

Furthermore, all models consistently achieved high recovery rates for proline and glycine. LM Design, in particular, excelled in predicting these residues, likely due to their unique dihedral angle distributions. This suggests that the models leverage structural information encoded in these distinct dihedral angle preferences during sequence design.

### Distinct model characteristics and training data influences

#### Distinct features of different models.

**AntiFold’s unique strengths:** AntiFold’s fine-tuning on antibody-specific datasets contributed to its superior performance in Fab antibodies, particularly in capturing the variability of H3 (S4 Table in S1 File). Its reduced reliance on antigen context further enhances its robustness for antigen-independent tasks. To address potential memorization concerns, we tested models with relaxed structures as templates, observing minimal impact on recovery rates for AntiFold. This indicates robustness to noise and reliance on structural features rather than memorization (S8 Fig in S1 File).

**LM-Design’s adaptability:** LM-Design’s integration of protein language models allowed it to generalize effectively across both Fab and VHH antibodies. Its balanced performance across CDRs highlights its potential for designing antibodies with diverse structures.

**Challenges for general protein models:** ESM-IF and ProteinMPNN, trained primarily on general protein datasets, struggled to capture the unique sequence-structure relationships of antibodies. These models exhibited biases toward overrepresented residues and failed to effectively design variable regions like H3.

**Fig 11:**
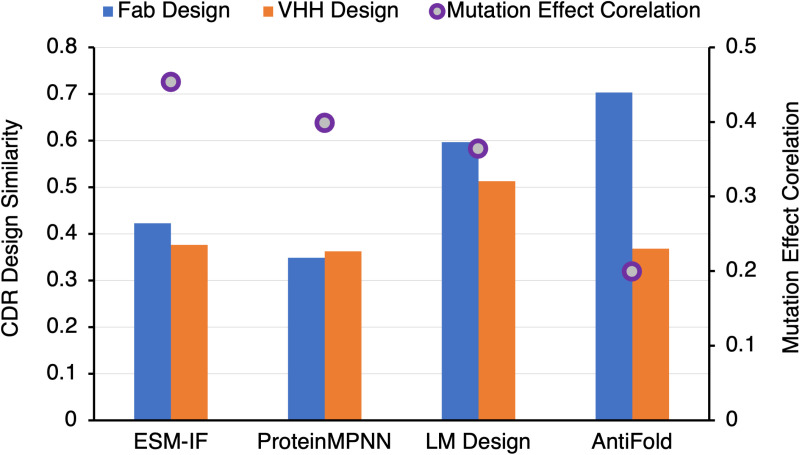
Design similarity and mutation effect correlation across inverse folding design models. This figure summarizes key metrics for inverse folding design models. The BLOSUM62 similarity to wild-type (WT) sequences is shown for Fab designs (blue bars) and VHH designs (orange bars) on the left y-axis, while the mutation effect correlation (purple cycles) is plotted on the right y-axis. All metrics were calculated as the average across all data points in the benchmarking dataset, highlighting model performance across sequence similarity and predictive accuracy for mutational effects.

We summarized the CDR sequence design capability for both Fab and VHH, and mutational fitness prediction capability of different models in Fig 11.

#### Antibody-specific features.

The performance of inverse folding models is heavily influenced by the specificity of their training datasets. AntiFold and LM-Design consistently demonstrated higher sequence recovery rates for antibody-specific features, particularly in conserved regions such as H1 and the C-terminus of H3. This suggests these models effectively learned antibody-specific structural and sequence motifs during training. For example, AntiFold’s fine-tuning on antibody-specific datasets, such as SAbDab and modeled structures from OAS, enabled it to capture the unique sequence-structure relationships present in antibody frameworks. Conversely, LM-Design benefits from its integration of a protein language model, which likely helps refine its antibody-specific predictions during inference. Despite being trained on general protein datasets, the additional structural encoder enables LM-Design to incorporate antibody-specific features effectively.

### Limitations of general protein data

Models such as ProteinMPNN and ESM-IF, trained on general protein datasets, showed limitations in capturing antibody-specific nuances. For example, these models often struggled to generate sequences for H3, which is highly variable and critical for antigen binding. This limitation likely arises from their reliance on datasets dominated by non-antibody proteins, where such structural diversity is less prevalent.

Furthermore, general protein training data may lead to biases in sequence design. For instance, ProteinMPNN and ESM-IF exhibited overrepresentation of high-frequency amino acids (e.g., serine, glycine) that are common across general proteins but less contextually appropriate for certain antibody regions.

The differences in model performance highlight the need for curated training datasets to improve antibody-specific design. AntiFold’s poor performance on VHH sequences underscores this issue. As VHH antibodies were largely absent from its training set, the model struggled to generate sequences for these structures, demonstrating reduced generalizability across antibody subclasses.

To address such limitations, future models should incorporate broader datasets that include VHH antibodies and antigen-antibody complexes. Additionally, integrating functional data such as binding affinities and mutational landscapes could enhance models’ ability to prioritize residues critical for binding, particularly in H3 regions.

Finally, the reliance on antigen information also varies among models. AntiFold, for instance, displayed minimal dependence on antigen structure, focusing primarily on the CDR loop itself. While this improves its robustness in antigen-independent tasks, it may reduce its utility for designing antigen-specific interactions, a critical consideration for therapeutic applications.

### Future directions for antibody inverse folding

While antibody inverse folding models have demonstrated promising progress, there remains significant potential for improvement. Below, we expand on five key areas for advancing this field, with practical examples and potential impacts highlighted.

**Enhanced training data:** Models trained on general protein data may not fully capture antibody-specific nuances, particularly in H3 design as shown in this study. Curating larger, more diverse antibody-specific datasets, including VHH sequences and broader antigen-antibody complexes, could improve model performance and generalizability.

**Integration of functional data:** Incorporating functional data, such as binding affinities and mutational effects, could enhance model accuracy and informativeness, e.g., the model could focus more on antibody-antigen interactions when designing the interface residues. This could be achieved through multi-task learning or by developing models that explicitly consider both structural and functional constraints.

**Improved generalization:** Addressing limitations in generalizing to different antibody types and structural contexts is crucial. Techniques such as transfer learning, data augmentation, and incorporating diverse structural templates during training could improve model generalization.

**Comprehensive evaluation metrics:** Developing more comprehensive evaluation metrics that capture the functional and biophysical properties of designed antibodies is essential. This could involve incorporating experimental validation, biophysical simulations, and machine learning-based predictors of antibody properties.

**Mitigation of biases:** Addressing potential biases in the models, such as overrepresentation of certain amino acids, is necessary. Adjusting training data distributions, incorporating regularization methods, or developing models that explicitly account for amino acid usage biases could mitigate these biases.

By addressing these areas, future research can drive the development of more accurate, generalizable, and robust inverse folding models, facilitating the design of novel antibodies with desired properties for therapeutic and biotechnological applications. Generative LLMs for antibody design have the potential to accelerate drug discovery and design by delivering better and faster lead candidates, e.g., desired epitope, no liabilities that could be bespoke to individual patients needs and thereby enable the next generation of therapeutics.

## Supporting information

S1 File Supplementary. (PDF)
