# Supplementary material for: Benchmarking inverse folding models for antibody CDR sequence design
Source: PLoS One. 2025 Jun 4;20(6):e0324566. doi: 10.1371/journal.pone.0324566 (PMC12136355; doi:10.1371/journal.pone.0324566)
Supplement: S1 File — (PDF) [file pone.0324566.s001.pdf]

Supplementary Information for

Benchmarking inverse folding models for antibody CDR sequence design

Yifan Li[1], Yuxiang Lang[1], Chenrui Xu[1], Yi Zhou[1], Ziwei Pang[1], Per Jr. Greisen[1,2 *]

[1] BioMap Research, JD Technology Building, 76 Zhichun Road, Haidian District, Beijing, China

[2] BioMap Research, 101 Jefferson Drive, Menlo Park, CA 94025, USA

*Correspondence:

Email: per@biomap.com

Supplementary Data

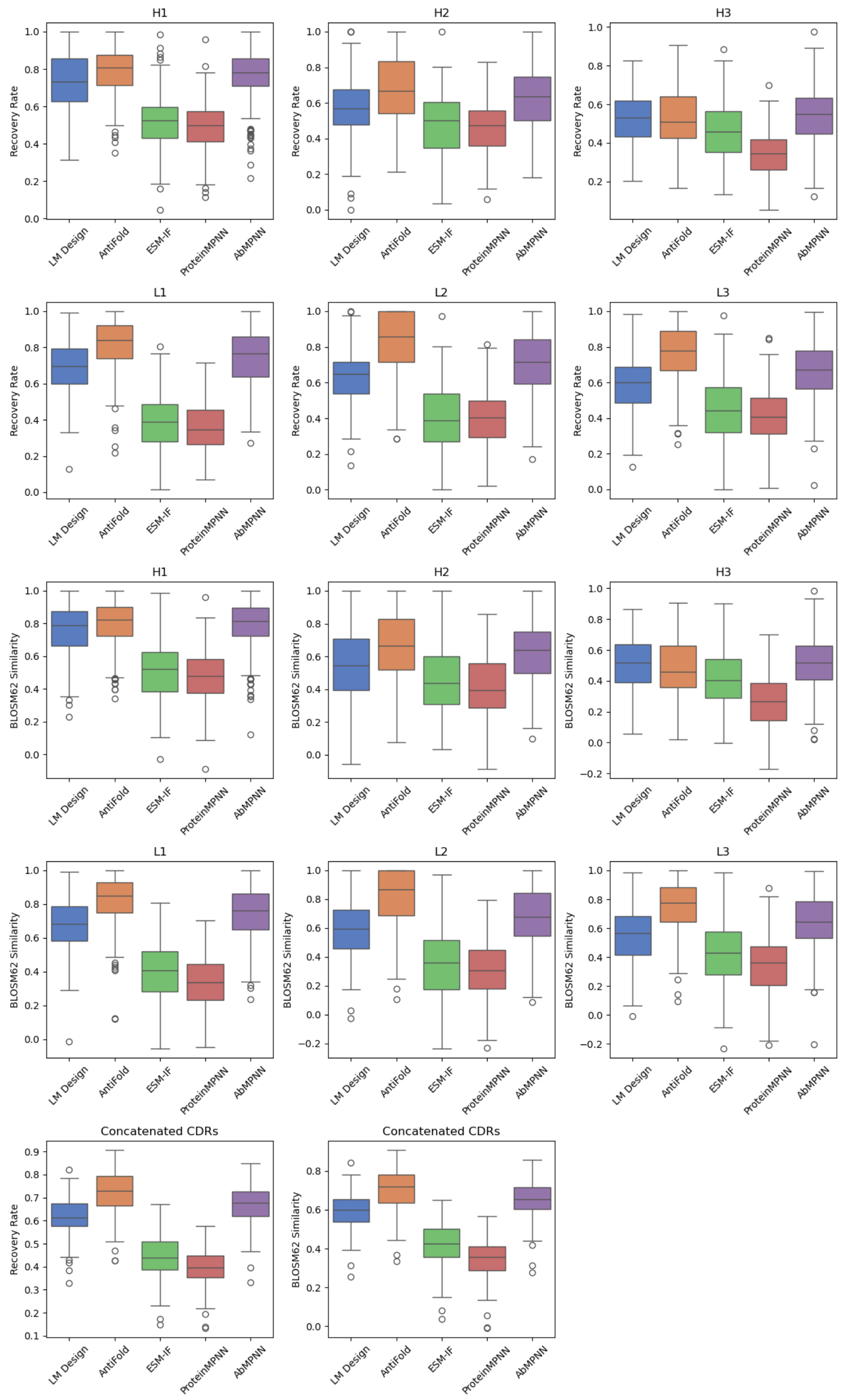

Supplementary Figure 1: Sequence Identity and Similarity for Fab CDRs. Box plots comparing

sequence identity and similarity for individual and concatenated CDRs designed with different methods for Fab, evaluated on 203 Fab crystals from SABDAB. CDRs defined by the Chothia numbering scheme.

Supplementary Figure 2

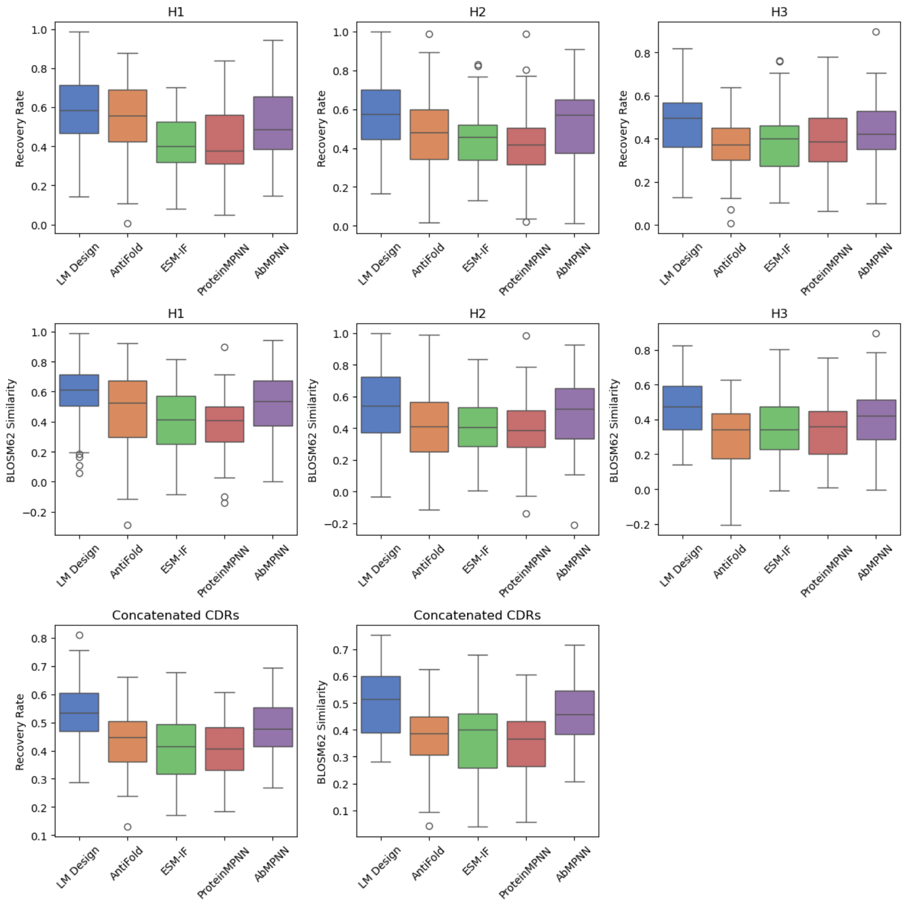

Supplementary Figure 2. Sequence Identity and Similarity for VHH CDRs. Box plots comparing sequence identity and similarity for individual and concatenated CDRs designed with different methods for VHH, evaluated on 61 VHH crystals from SABDAB. CDRs defined by the Chothia numbering scheme.

Supplementary Figure 3

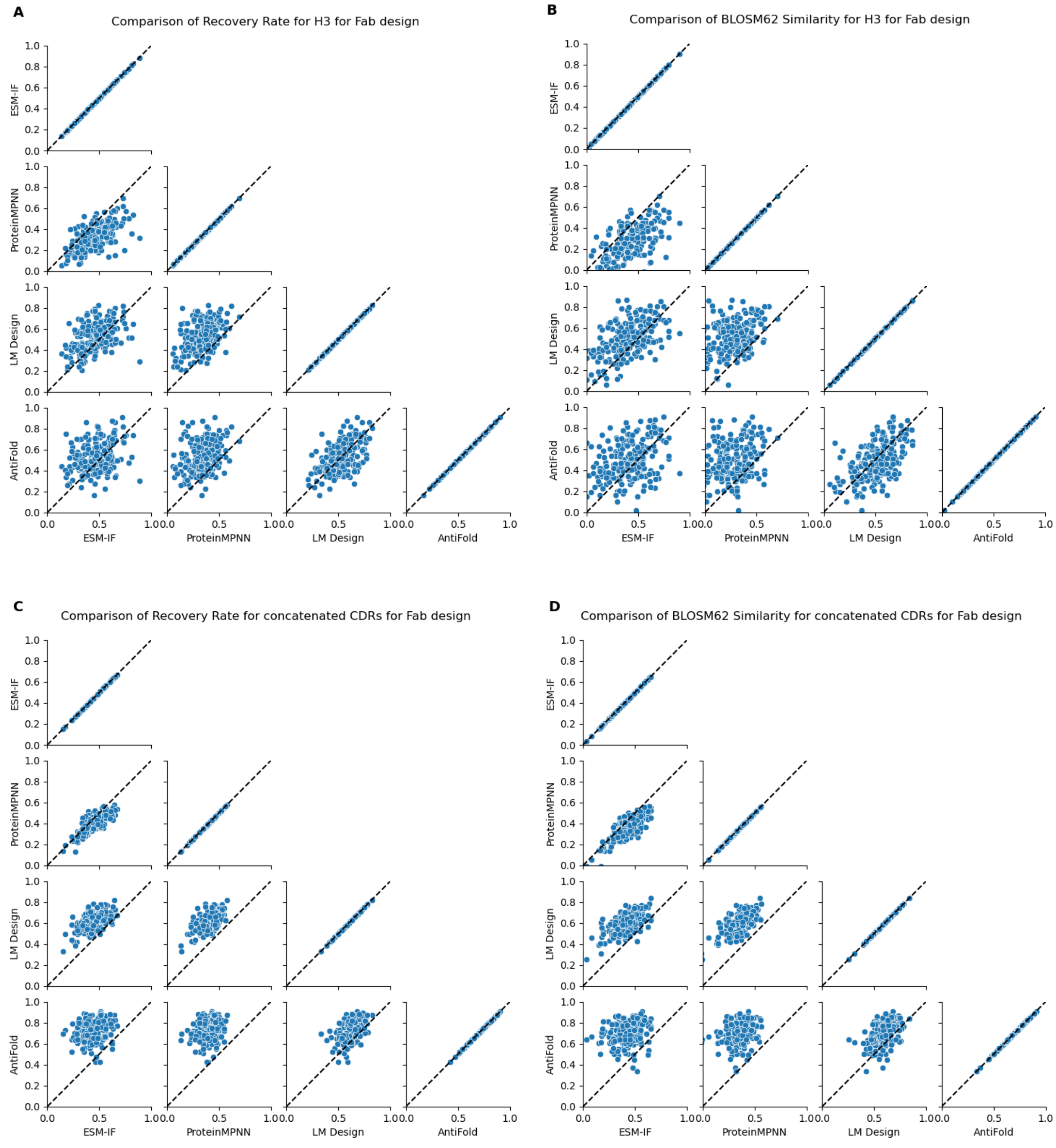

Supplementary Figure 3. Correlation of Fab CDR Sequence Design Models for (A) Recovery for H3, (B) BLOSM62 similarity for H3, (C) Recovery for concatenated CDRs, (D) BLOSM62 similarity for concatenated CDRs. Scatterplot matrix showing correlations between different

methods for Fab CDR sequence design, evaluated on 203 Fab crystals from SABDAB. Each dot represents a PDB crystal. CDRs defined by the Chothia numbering scheme.

Supplementary Figure 4

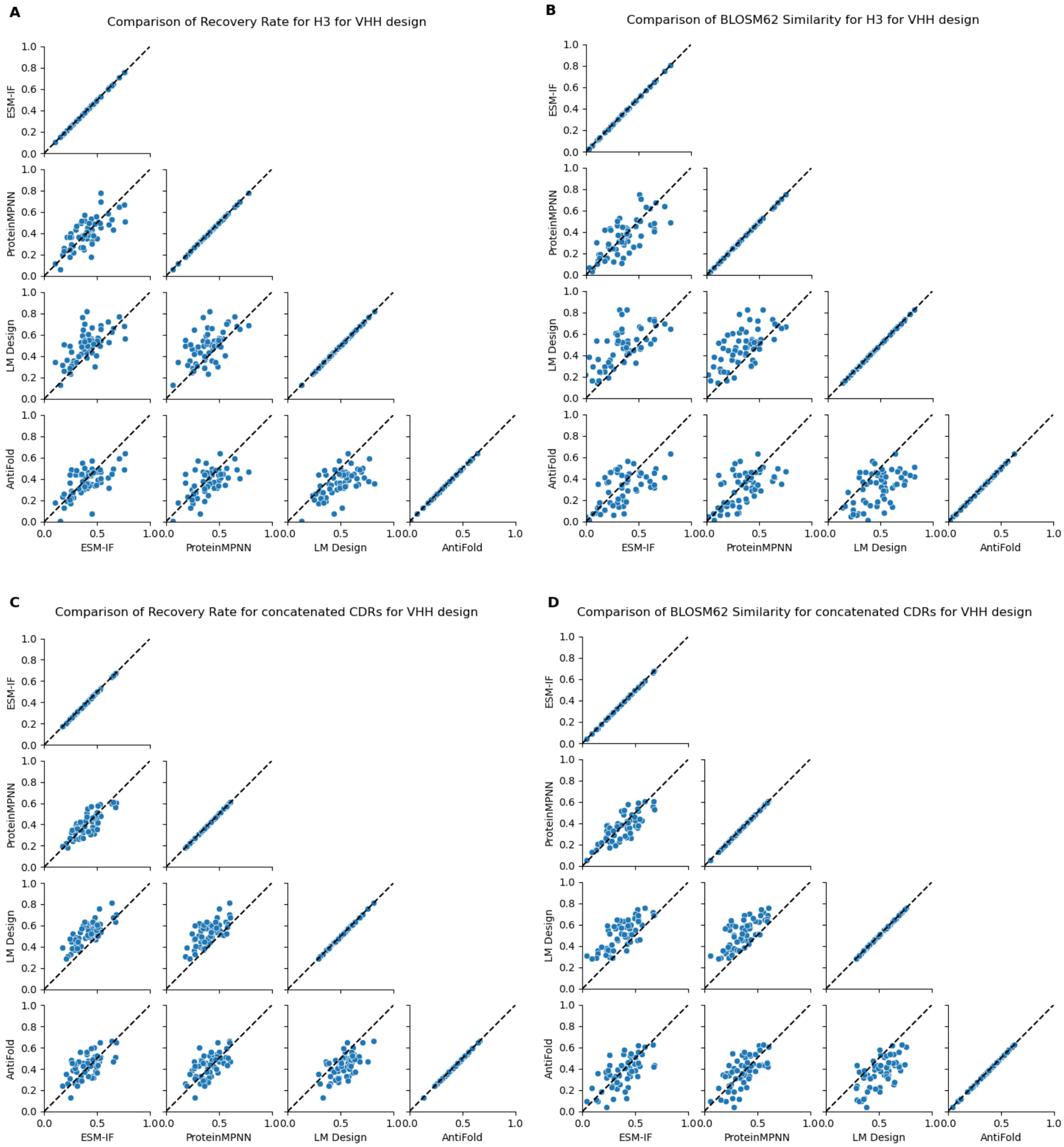

Supplementary Figure 4. Correlation of VHH CDR Sequence Design Models for (A) Recovery for H3, (B) BLOSUM62 similarity for H3, (C) Recovery for concatenated CDRs, (D) BLOSUM62 similarity for concatenated CDRs. Scatterplot matrix showing correlations between different methods for VHH CDR sequence design, evaluated on 61 VHH crystals from SABDAB. Each dot

represents a PDB crystal structure. CDRs defined by the Chothia numbering scheme.

Supplementary Figure 5

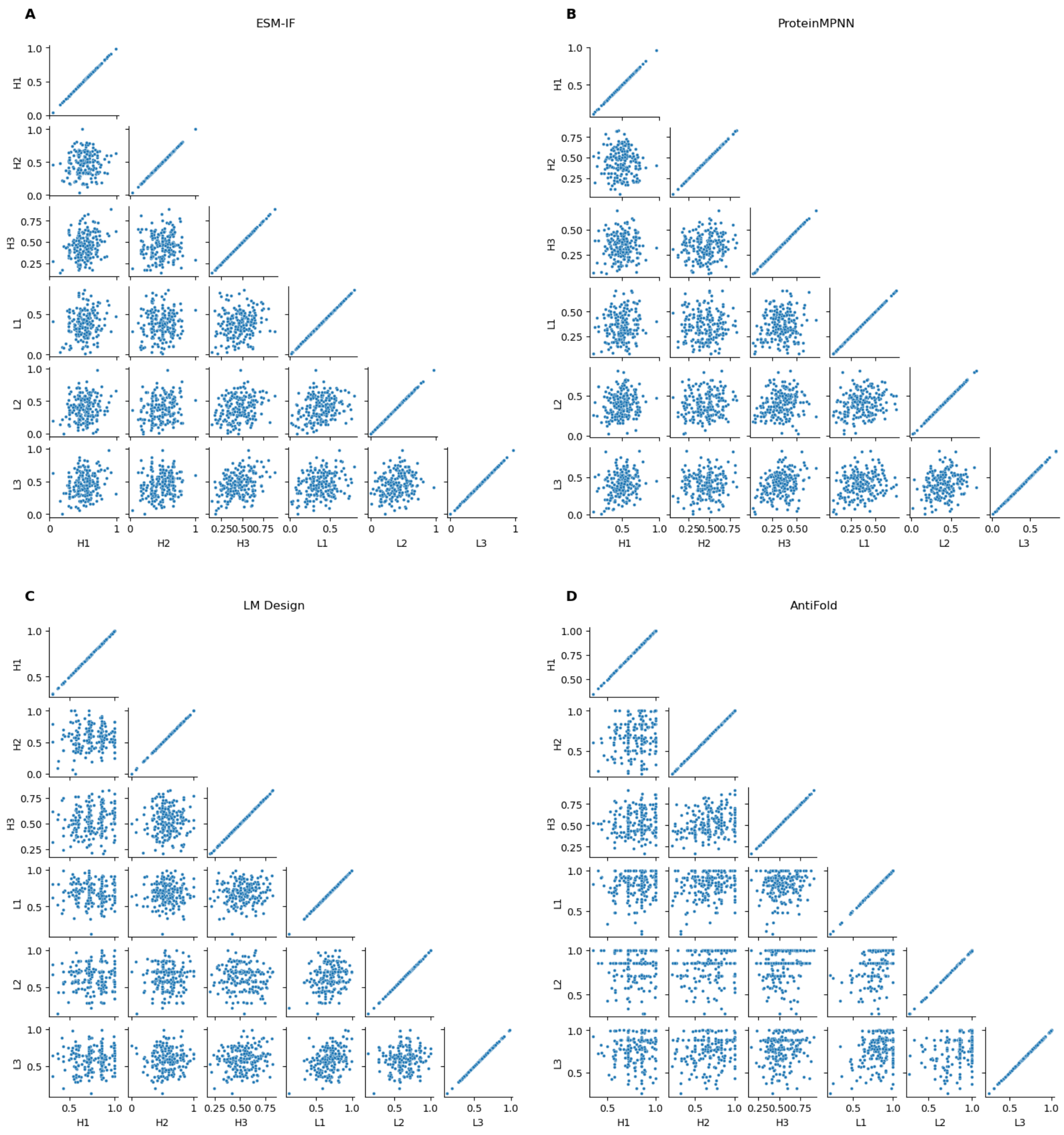

Supplementary Figure 5. Pair plots showing the correlation of recovery rates between different CDR regions across various PDB structures for four sequence design models: (A) ESM-IF, (B) ProteinMPNN, (C) LM Design, and (D) AntiFold. Each sub-figure represents a distinct design

model, with each dot corresponding to the recovery rate for a single PDB structure.

Supplementary Figure 6

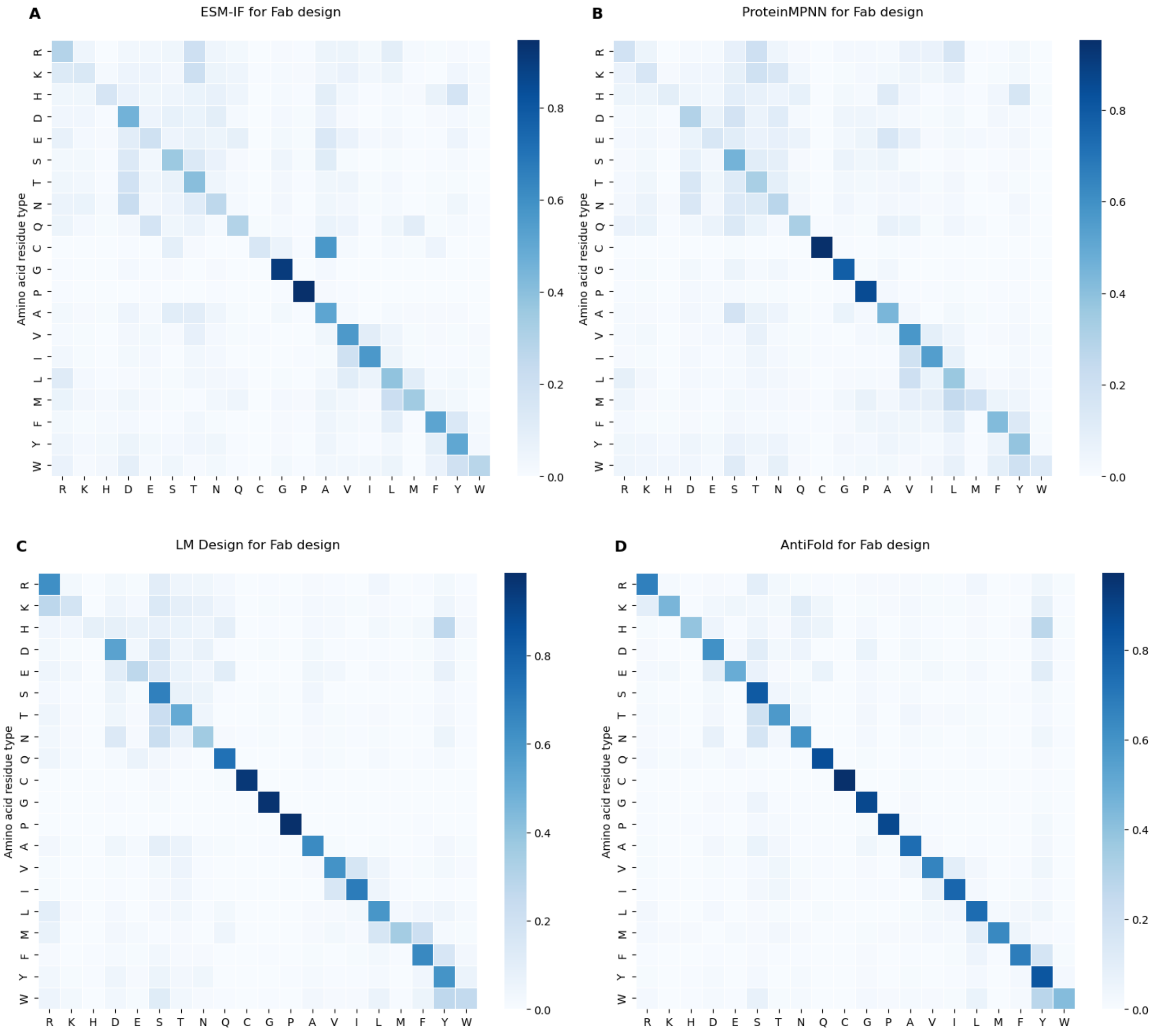

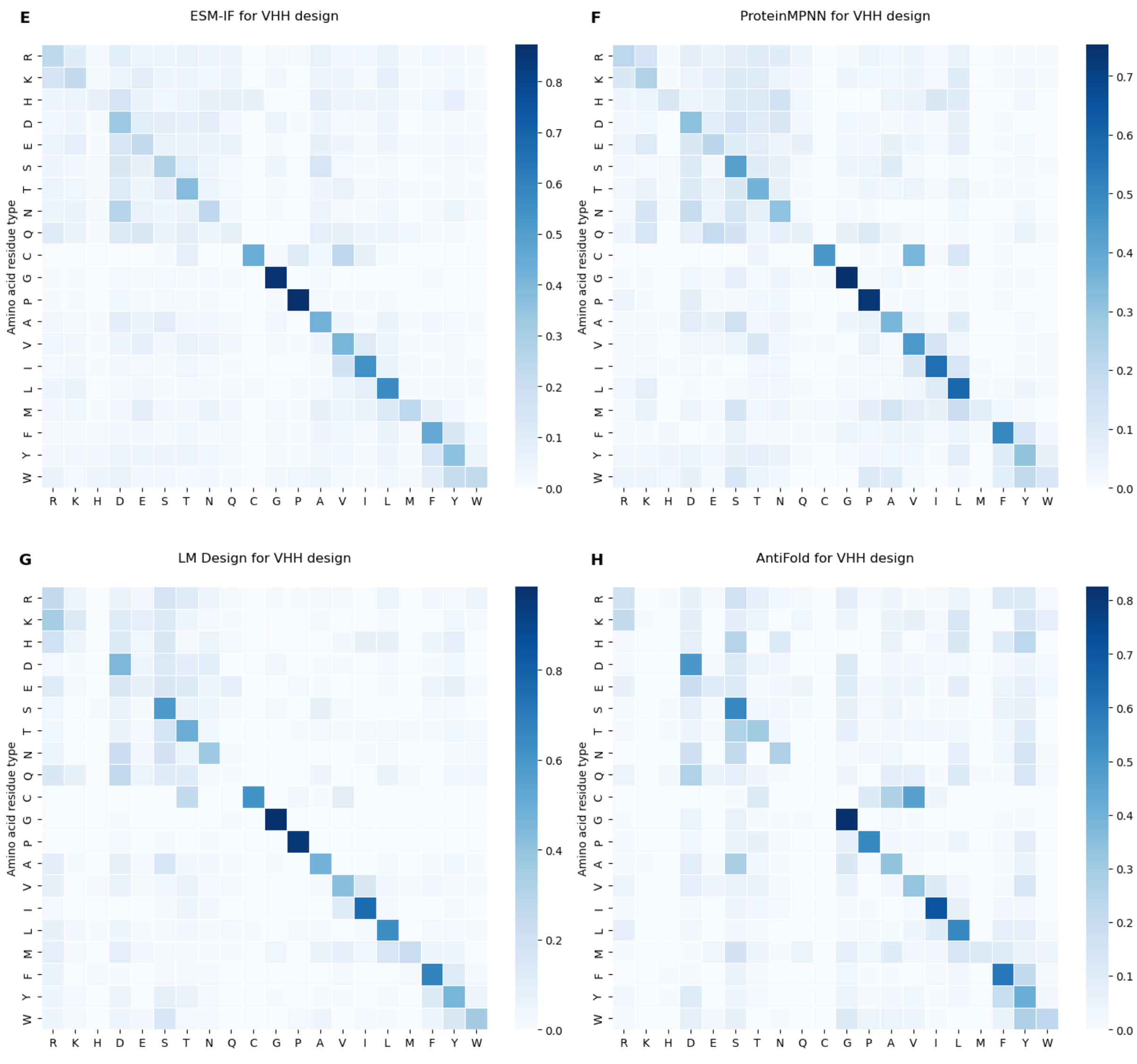

Supplementary Figure 6. Confusion Matrix for Residue Type Prediction for (A) ESM-IF for Fab design, (B) ProteinMPNN for Fab design, (C) LM Design for Fab design, (D) AntiFold for Fab design, (E) ESM-IF for VHH design, (F) ProteinMPNN for VHH design, (G) LM Design for VHH design, (H)AntiFold for VHH design. Confusion matrices showing the accuracy of residue type prediction for different models.

Supplementary Figure 7

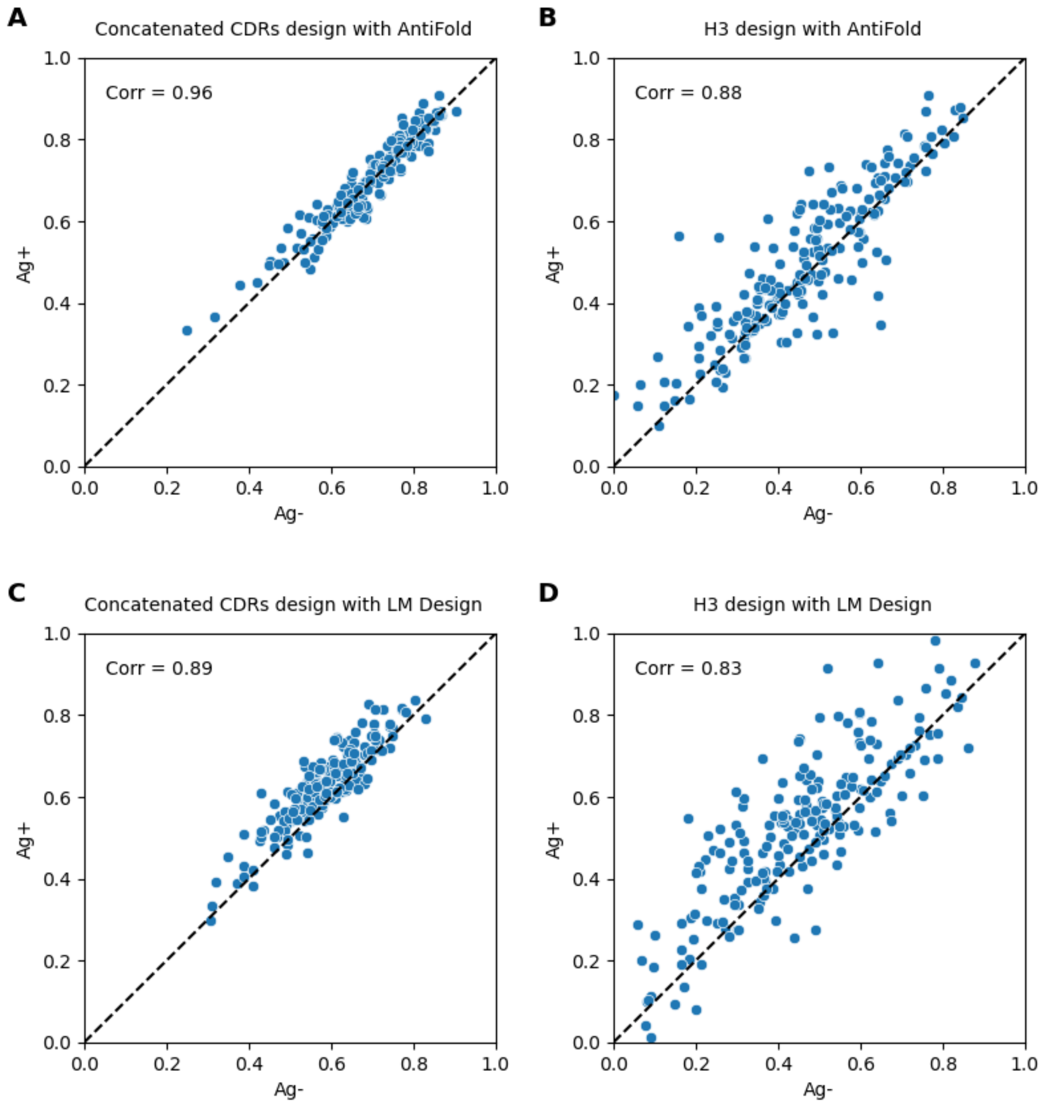

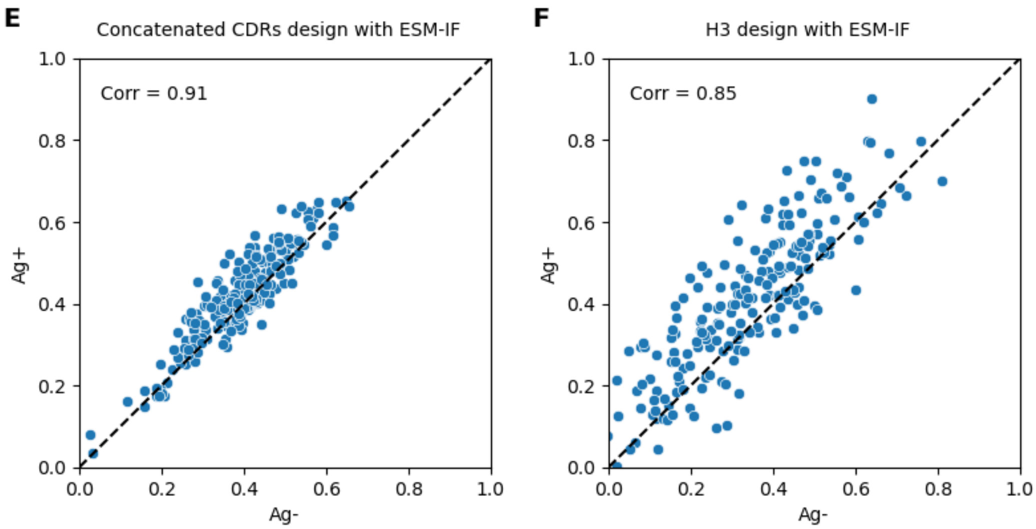

Supplementary Figure 7. Impact of Antigen Chain on Fab Sequence Design Similarity. Scatter plots comparing sequence design similarity with antigen (Ag+) and without antigen (Ag-) present in the structure. Results are shown for different models: (A) concatenated CDRs design with AntiFold, (B) H3 design with AntiFold, (C) concatenated CDRs design with LM Design, (D) H3 design with LM Design, (E) concatenated CDRs design with ESM-IF, and (F) H3 design with ESM-IF. Distinct distributions indicate the influence of antigen presence on sequence design outcomes. Statistical comparisons were performed using Pearson correlation analysis.

Supplementary Figure 8

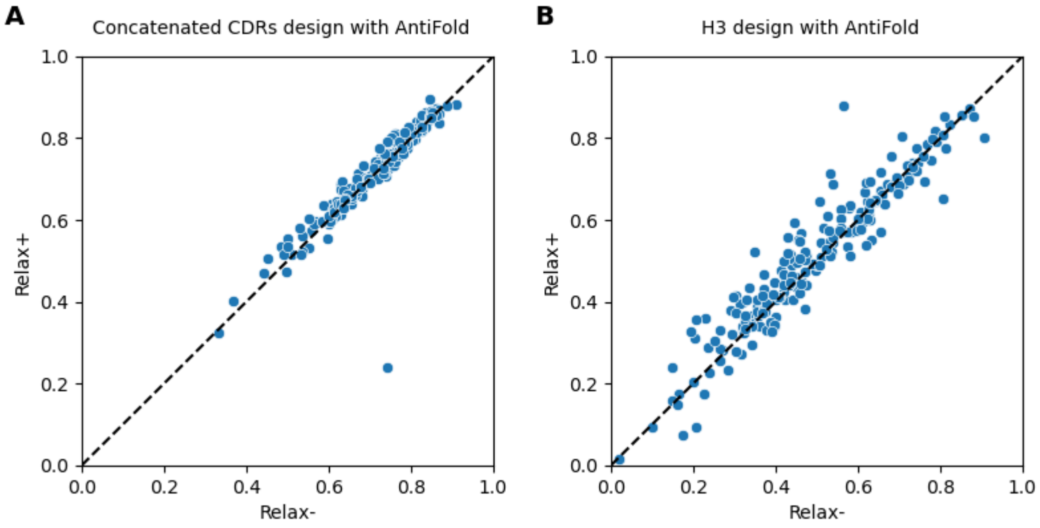

Supplementary Figure 8. Effect of Relaxation on Design Similarity. Scatter plots and histograms showing the distribution of sequence design similarity with (Relax+) and without (Relax-) applying coordinate relaxation to the input structures. Relax- refers to sequence design similarity using the original, unrelaxed input structures, while Relax+ represents sequence design similarity after applying coordinate relaxation. Coordinate relaxation was performed using the Rosetta FastRelax protocol to minimize the input structure (see Methods section for details). Analysis includes various design models, highlighting how structural minimization influences design similarity.

Supplementary Figure 9

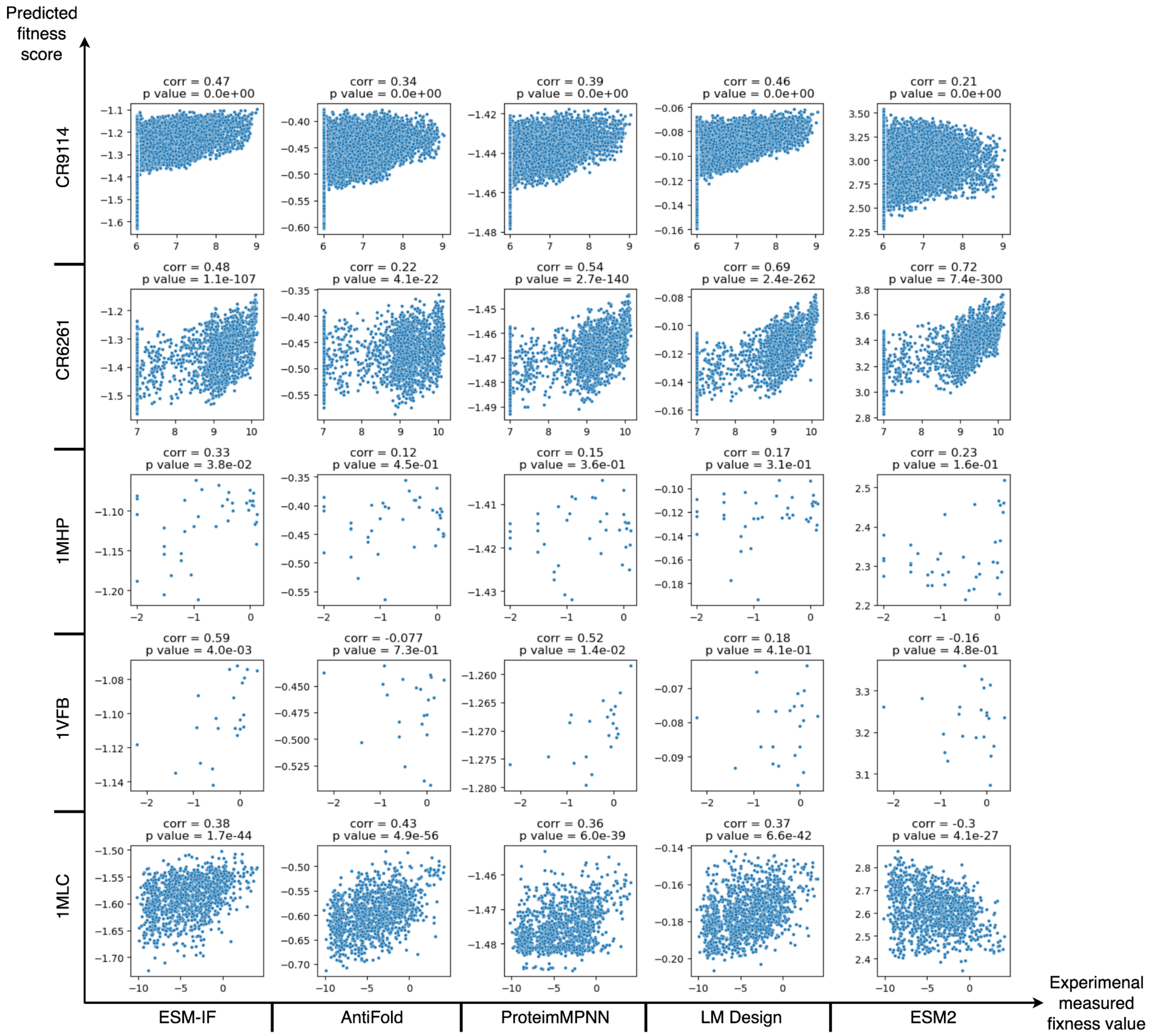

Supplementary Figure 9. Scatter plots showing the relationship between model-predicted fitness scores and experimentally measured fitness scores across different models and evaluation datasets. Each row corresponds to a different dataset (indicated by the labels on the y-axis), while each column represents a different model (labeled on the x-axis). In each subplot, the x-axis denotes the experimental fitness scores, and the y-axis represents the fitness scores predicted by the respective

model. The Spearman correlation coefficient (corr) and statistical significance (p-value) are displayed at the top of each subplot.

Supplementary Figure 10

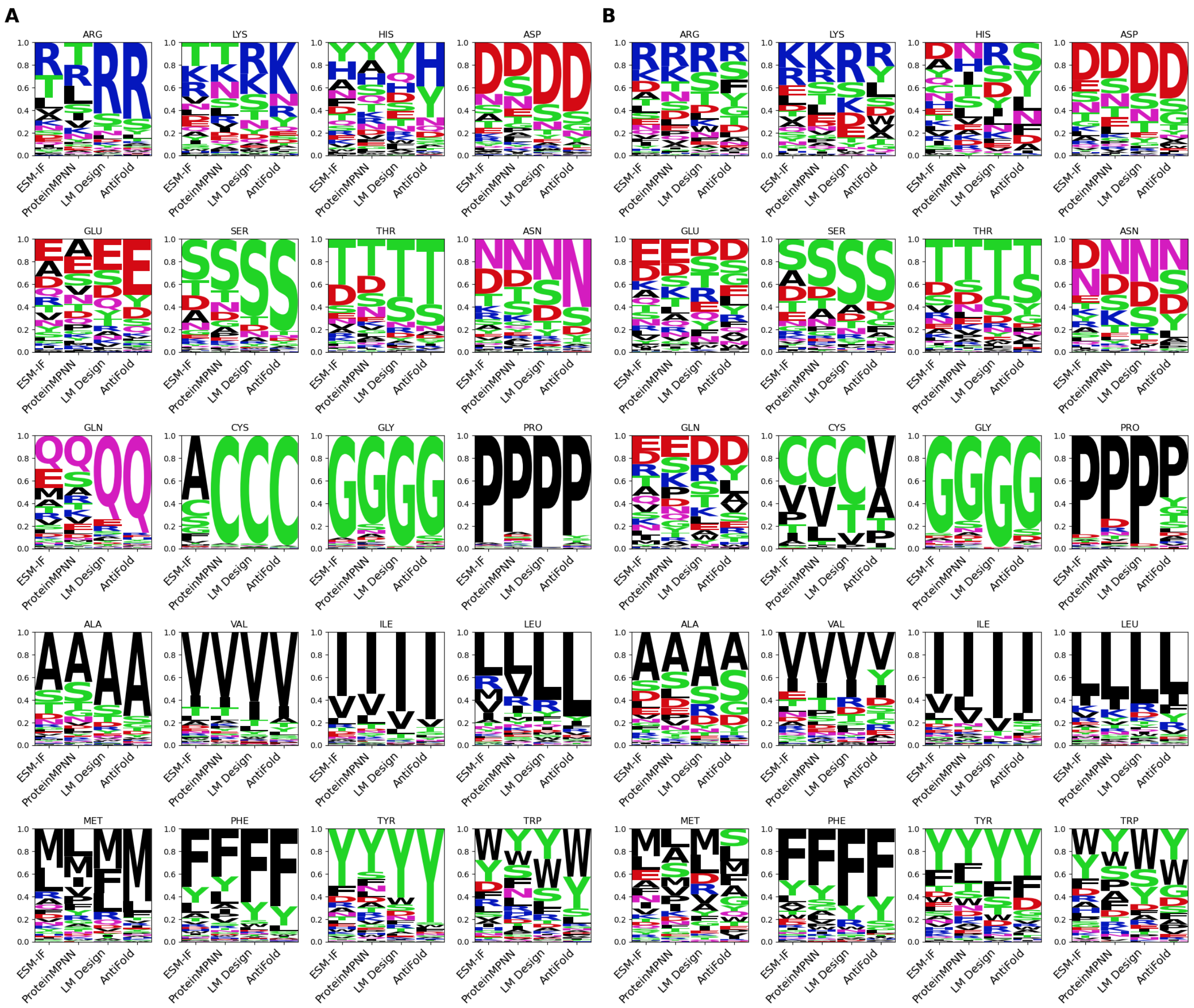

Supplementary Figure 10. Logo Plot of predicted residue frequencies for (A) Fab design, and (B) VHH design. Logo plots showing the frequency of predicted residue types for different sequence design models, based on all predicted CDRs, and grouped by their corresponding wild-type residue type. This visualization highlights residue preference patterns across models and their alignment with wild-type sequences.

Supplementary Figure 11

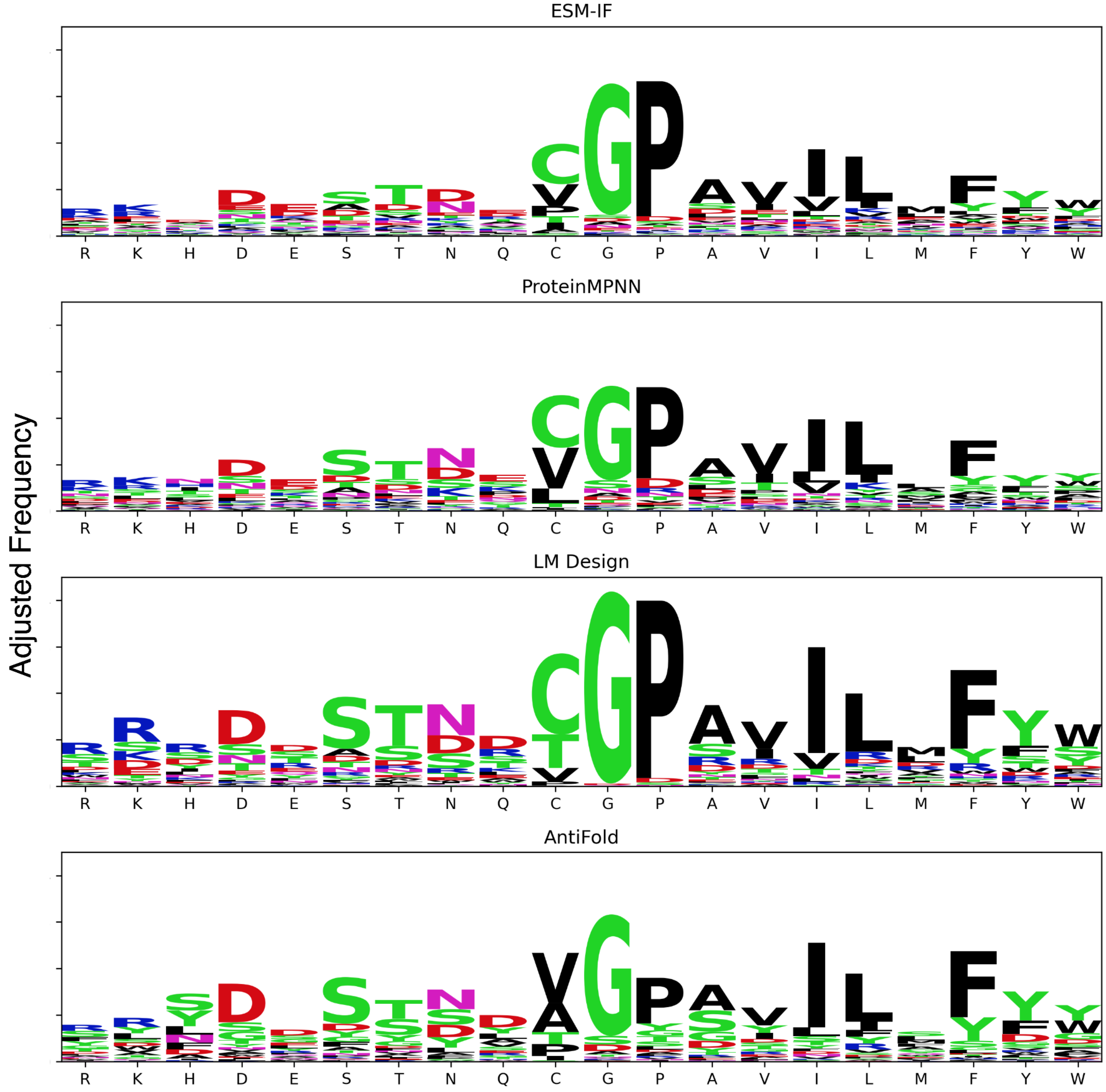

Supplement Figure 11. Logo plots showing the predicted amino acid frequencies for each wild-type (WT) residue in the CDRs of VHH structures. WT residues are ordered by descending frequency. The height of each letter indicates the relative frequency of predicted amino acids, highlighting each model's substitution tendencies. The overall height of each column indicates the conservativeness of the design for each type of residue. The size of each residue printed in a logo is determined by multiplying the frequency of that base

by the total information at that position.

Supplementary Figure 12

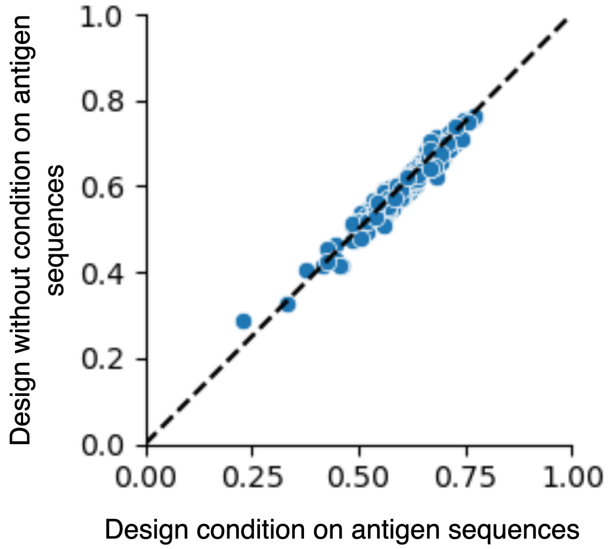

Supplementary Figure 12. Effect of antigen sequence conditioning on Design Similarity. Scatter plots showing sequence design similarity with and without conditioning on antigen sequences using the LM Design model for Fab CDR design.

Supplementary Figure 13

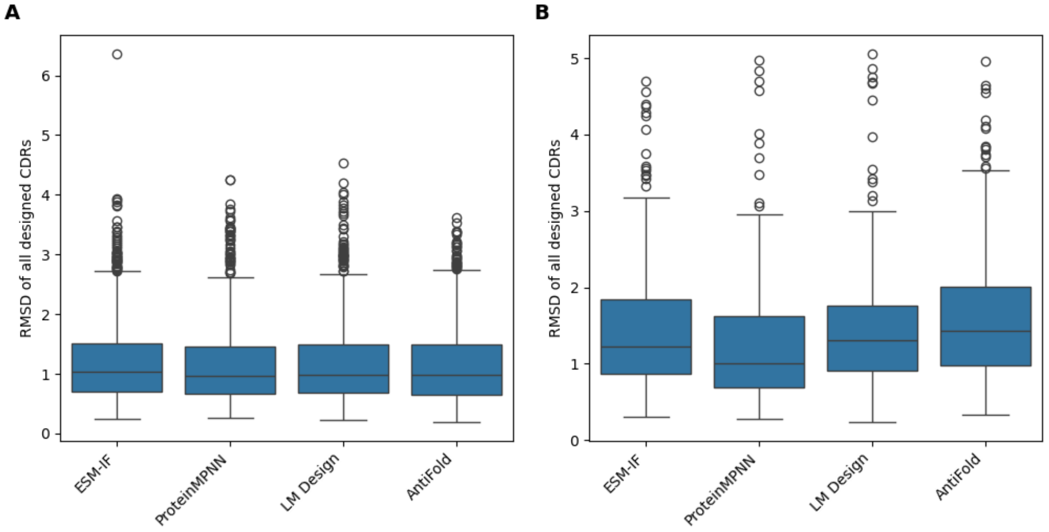

Supplementary Figure 13. RMSD between Refolded and Template Structures. Panel A shows the re-folded protein and the design template for Fab and Panel B shows VHH designs re-folded and RMSD measured.

Supplementary Table 1

Table 1a

Protein sequence design recovery rate for PDB ID: 8TFL for different CDR regions. This table, together with Figure 7 illustrates the design example for PDB ID: 8TFL.

Method	Concat CDRs	H1	H2	H3	L1	L2	L3
LM Design	0.558	0.904	0.607	0.368	0.573	0.429	0.595
AntiFold	0.677	0.893	0.662	0.443	0.641	0.851	0.75
ESM-IF	0.434	0.461	0.528	0.347	0.387	0.514	0.465
ProteinMPNN	0.372	0.299	0.505	0.309	0.256	0.479	0.499

Table 1b

Statistical Significance of Pairwise Model Comparisons. Model_1 and Model_2 specify the two models being compared. The significant values were calculated by Wilcoxon rank-sum test.

Model_1	Model_2	Concat CDRs	H1	H2	H3	L1	L2	L3
ESM-IF	AntiFold	7.37E-35	6.66E-36	1.93E-28	8.36E-29	2.35E-39	2.28E-38	3.64E-41
ESM-IF	LM Design	1.37E-34	6.13E-37	1.46E-11	3.44E-02	1.25E-35	1.77E-09	2.76E-27
ESM-IF	ProteinMPNN	2.07E-26	3.53E-28	1.38E-03	7.21E-08	5.26E-25	6.49E-02	1.42E-06

LM Design	AntiFold	6.27E-35	6.15E-01	3.14E-08	4.71E-13	4.48E-25	5.90E-44	9.52E-41
ProteinMPNN	AntiFold	4.93E-35	1.22E-37	2.86E-36	3.00E-32	4.55E-39	1.82E-38	1.48E-44
ProteinMPNN	LM Design	7.37E-35	2.18E-38	8.84E-19	1.16E-07	1.25E-36	3.91E-06	7.84E-27

Supplementary Table 2

All pdb information in the Fab evaluation dataset, refer to github repository for detail

Supplementary Table 3

All pdb information in the VHH evaluation dataset, refer to github repository for detail

Supplementary Table 4

Mean value for all evaluated Fab across different CDR regions using various models.

	Design Recovery							Design Similarity						
Model	H1	H2	H3	L1	L2	L3	Concat CDRs	H1	H2	H3	L1	L2	L3	Concat CDRs
AbMPNN	0.762	0.628	0.545	0.749	0.708	0.666	0.67	0.784	0.627	0.514	0.748	0.676	0.643	0.653
AntiFold	0.787	0.679	0.53	0.818	0.836	0.765	0.726	0.799	0.655	0.49	0.823	0.816	0.747	0.703
ESM-IF	0.518	0.48	0.459	0.382	0.393	0.452	0.444	0.512	0.454	0.406	0.398	0.343	0.424	0.423
LM Design	0.745	0.574	0.527	0.685	0.631	0.588	0.62	0.761	0.552	0.506	0.679	0.584	0.552	0.597
ProteinMPNN	0.496	0.453	0.339	0.357	0.402	0.406	0.398	0.48	0.414	0.259	0.335	0.315	0.352	0.349

Supplementary Table 5

Mean value for all evaluated VHH across different CDR regions using various models.

	Design Recovery				Design Similarity			
Model	H1	H2	H3	Concat CDRs	H1	H2	H3	Concat CDRs
AbMPNN	0.514	0.516	0.443	0.475	0.517	0.493	0.41	0.454
AntiFold	0.527	0.466	0.367	0.432	0.469	0.411	0.299	0.368
ESM-IF	0.419	0.44	0.396	0.41	0.399	0.418	0.349	0.376
LM Design	0.594	0.562	0.487	0.528	0.585	0.538	0.473	0.513
ProteinMPNN	0.42	0.422	0.396	0.405	0.391	0.395	0.342	0.362

Supplementary Table 6

Paired sample t-tests were conducted to compare the performance of different Fab design methods.

methods	p value for BLOSM62 similarity for concatenated CDRs	p value for recovery for concatenated CDRs
ESM-IF vs ProteinMPNN	7.49E-37	8.76E-24
ESM-IF vs LM Design	1.93E-67	5.59E-77
ESM-IF vs AntiFold	2.53E-73	6.05E-84
ProteinMPNN vs LM Design	4.95E-106	1.12E-106
ProteinMPNN vs AntiFold	8.77E-99	4.34E-103
LM Design vs AntiFold	1.93E-35	9.39E-42

Supplementary Table 7

Paired sample t-tests were conducted to compare the performance of different VHH design methods

methods	p value for BLOSM62 similarity for concatenated CDRs	p value for recovery for concatenated CDRs
ESM-IF vs ProteinMPNN	0.22092741	0.55634246

ESM-IF vs LM Design	4.67E-16	3.66E-18
ESM-IF vs AntiFold	0.58528516	0.09287898
ProteinMPNN vs LM Design	1.22E-17	2.06E-16
ProteinMPNN vs AntiFold	0.70373584	0.03171469
LM Design vs AntiFold	7.23E-14	5.43E-11

Supplementary Method

Rosetta FastRelax with coordinate constraints xml file:

```xml
<ROSETTASCRIPTS>
  <SCOREFXNS>
    <ScoreFunction name="sfxn" weights="ref2015_cart">
      <Reweight scoretype="cart_bonded" weight="1.5"/>
      <Reweight scoretype="coordinate_constraint" weight="1"/>
    </ScoreFunction>
  </SCOREFXNS>

  <MOVERS>
    <AddConstraints name="add_csts" >
      <CoordinateConstraintGenerator name="gen_my_csts"
        bounded="0" ca_only="0" sidechain="1" sd="0.5" />
    </AddConstraints>
    <FastRelax name="FastRelax" scorefxn="sfxn" repeats="3" batch="false" ramp_down_constraints="false" cartesian="true" bondangle="false" bondlength="false" min_type="dfpmin_armijo_nonmonotone" />
  </MOVERS>

  <PROTOCOLS>
    <Add mover="add_csts"/>
    <Add mover="FastRelax"/>
  </PROTOCOLS>

  <OUTPUT />

</ROSETTASCRIPTS>
```
